# Within‐Island Diversification Generates Lineage‐Specific Climate Vulnerability: Insights From the Taiwan‐Endemic Formosan Duke

**DOI:** 10.1111/eva.70309

**Published:** 2026-08-02

**Authors:** Zong‐Yu Shen, Yu‐Feng Hsu, Masaya Yago, Chih‐Chien Lu, Ming‐Yu Chen, Wei‐Yun Chen, Tzong‐Han Lin, Jen‐Pan Huang, Li‐Wei Wu

**Affiliations:** ^1^ The University Museum The University of Tokyo Tokyo Japan; ^2^ Department of Global Agricultural Sciences, Graduate School of Agricultural and Life Sciences The University of Tokyo Tokyo Japan; ^3^ Department of Life Science National Taiwan Normal University Taipei Taiwan; ^4^ Taichung Municipal Shuang‐Wen Junior High School Taichung Taiwan; ^5^ Department of Life Science Tunghai University Taichung Taiwan; ^6^ Biodiversity Research Center, Academia Sinica Taipei Taiwan; ^7^ Department of Entomology National Taiwan University Taipei Taiwan; ^8^ Center for Ecology and Environment Tunghai University Taichung Taiwan

**Keywords:** central mountain range, climate change, conservation, *Euthalia*, genetic offset

## Abstract

Global climate change disproportionately threatens island endemics because their restricted ranges and limited dispersal opportunities constrain their ability to shift their distributions. Therefore, when developing conservation strategies for species, it is crucial to incorporate population genomics data with ecological information to evaluate resilience and forecast vulnerability. However, genomic case studies that explicitly link these factors in island endemics remain scarce. Here, we examine population genetic differentiation of the Formosan Duke (*Euthalia formosana*), an endemic butterfly in Taiwan, to evaluate its potential vulnerabilities under future climate conditions. Our results reveal that the combined effects of topographic barriers, historical connectivity, and climatic heterogeneity can drive rapid diversification, even in species with strong flight ability. The Central Mountain Range has shaped pronounced genetic structure, separating eastern and western lineages, with diversification stronger in the western lineage. This within‐island diversification appears to be shaped by environmental gradients, enhancing persistence in local habitats while reducing a lineage's niche breadth and resulting in different lineage‐specific climate vulnerability. Thus, we suggest that genomic approaches should be included to assess environmental constraints and to guide conservation planning on the persistence of lowland endemics such as the Formosan Duke. This framework also integrates the concepts of habitat restoration with lineage‐specific connectivity to enhance the species' genetic resilience while preserving its capacity for local adaptation. Moreover, this study provides an evidence‐based framework for insular biodiversity conservation under accelerating climate change.

## 1 Introduction

Global climate and environmental change in the Anthropocene are rapidly reshaping patterns of biodiversity worldwide (Walther et al. 2002; Parmesan and Yohe 2003; Parmesan 2006). Although the pace and scale of contemporary change are exceptional, climatic fluctuations themselves are not unprecedented in the history of life. Episodes of substantially warmer temperatures have occurred repeatedly, not only during the Miocene Thermal Maximum, but also throughout Quaternary interglacial periods (Zachos et al. 2001; Otto‐Bliesner et al. 2006). As a result, many extant lineages have likely encountered environmental analogs to projected future climates during their recent evolutionary history. Understanding how species responded to these past climatic challenges, whether through local adaptation, range shifts, or demographic change, can therefore provide critical information on their responses to ongoing and future climate change. Recent advances in evolutionary genomic approaches now allow direct access to the genetic legacies of these historical processes (Moritz and Agudo 2013; Prates et al. 2016; Román‐Palacios and Wiens 2020). Genome‐wide analyses make it possible to detect signatures of adaptation associated with specific climate variables, demographic change, and more importantly to infer when and how these processes occurred (Huang et al. 2024; Shen et al. 2025; Lee et al. 2025). Such findings provide a mechanistic understanding of resilience and vulnerability under rapid environmental change and have direct relevance for the development of effective, evidence‐based conservation strategies.

To understand both the adaptation to past climate changes and the resulting consequences for adaptive capacity under future conditions, we require a study system where recent divergence allows for the reconstruction of evolutionary histories across well‐defined environmental and geographic gradients. In this context, the island of Taiwan, located offshore of southeastern China, provides an excellent terrestrial example of recent speciation. Its biogeographic position at the interface between subtropical and tropical climatic zones generates steep environmental gradients and a wide diversity of ecological niches. The biota has been shaped by repeated land‐bridge connections to mainland China during Pleistocene glacial periods (Ali and Fritz 2025), as well as by extensive within‐island diversification driven by fine‐scale geographic barriers and local adaptation (Huang and Lin 2010, 2011; Lin et al. 2025). The high level of endemism in Taiwanese biota has long been hypothesized to arise from two non‐mutually exclusive mechanisms (Hsu 2003): (1) neo‐endemism, involving recent colonization followed by in situ divergence, and (2) paleo‐endemism, reflecting the long‐term persistence of relict lineages. Empirical evidence supports both processes: several plant and vertebrate species show deep genetic divergences from their relatives in southwestern China, which is consistent with paleo‐endemic refugial histories (He et al. 2018; Ye et al. 2023). In contrast, repeated mainland‐to‐island colonization, followed by ecological divergence across heterogeneous niches, supports neo‐endemism in groups such as *Scutellaria* (Chiang et al. 2012).

Butterflies represent one of the most suitable groups for investigating endemic diversification in Taiwan. Decades of intensive field surveys and taxonomic revisions have produced a comprehensive inventory of the butterfly fauna, documenting 358 resident species, of which approximately one‐sixth are endemic species and nearly half are endemic subspecies (Shirôzu 1960; Hsu et al. 2018a, 2018b, 2019, 2020, 2021). Approximately two‐thirds of the endemic butterflies are restricted to mid‐ and high‐elevation montane habitats, whereas most lowland species exhibit a more extensive distribution across East Asia (Shirôzu 1960; Hsu et al. 2018a, 2018b, 2019, 2020, 2021). However, anthropogenic disturbance in Taiwan is concentrated in low‐ to mid‐elevation landscapes, where land use and habitat fragmentation are most severe (Chen et al. 2019). These pressures have unintentionally altered lowland forest ecosystems and increasingly threaten the survival of butterflies that depend on these habitats, with particularly high extinction risk for endemic taxa in these habitats.

**FIGURE 1:**
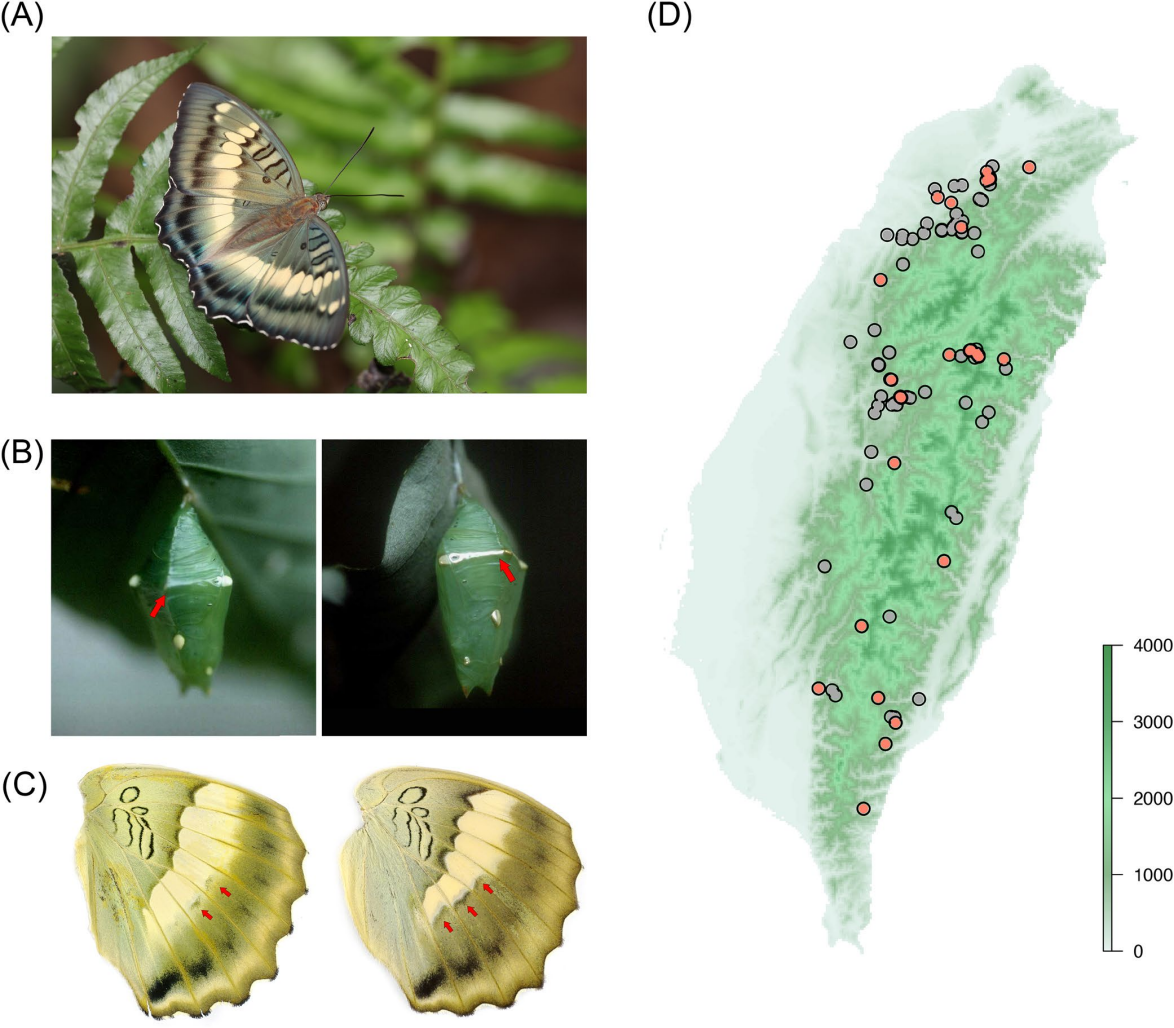
Morphological variation and geological distribution of *Euthalia formosana* in Taiwan. (A) Resting posture of a male *E. formosana* . (B) Geographic variation in pupal morphologies (continuous or broken silvery band). (C) Geographic variation in the ventral side of the adult hindwing. Red arrow indicates the black marginal area. (D) Historical occurrence records (grey) and sampling localities included in this study (red).

To examine the diversification processes of island endemics facing increasing climatic and anthropogenic changes in lowland regions (Hsieh et al. 1996), the Formosan Duke (*Euthalia formosana* Fruhstorfer, 1908; Figure 1A), a large endemic butterfly, provides a suitable candidate for evaluating the responses to climate change and habitat fragmentation. This butterfly is widely distributed across low‐ to mid‐elevation evergreen broad‐leaved forests. Its larvae primarily utilize host plants in the family Fagaceae, including *Quercus glauca* , *Q*. *longinux*, and *Lithocarpus hancei*, with occasional use of *Mallotus philippensis* (Euphorbiaceae) (Hsu et al. 2021). The broad geographic distribution and flexible host use of the Formosan Duke suggest strong dispersal capacity. However, despite this apparent dispersal potential, a previous study based on mitochondrial markers has detected distinct genetic structure among regions (Hsu 2006). In addition, our field observations reveal geographic phenotypic variation in both pupal and adult wing morphologies (Figure 1). Pupae from northern populations exhibit either a continuous or broken silvery band, whereas populations from other regions consistently display continuous bands (Figure 1B). Similarly, the extent of black marginal shading surrounding the white patches on the hindwing varies among regions (Figure 1C). The molecular and morphological differences suggest potential population differentiation and structure in this butterfly. These findings make it an ideal system for testing how geography, environment, and historical connectivity interact to shape diversification within an island. This framework also allows us to evaluate how differentiated lineages may respond to ongoing environmental changes.

In this study, we use double‐digest restriction‐site‐associated DNA sequencing (ddRADseq; Peterson et al. 2012) to investigate how geographic structure, environmental heterogeneity, and historical connectivity have shaped genome‐wide differentiation in the Formosan Duke, and whether these processes have generated lineage‐specific climate vulnerability. Specifically, we address three questions: (1) how major geographic features of Taiwan, particularly the Central Mountain Range (CMR) and lowland habitat fragmentation, structure population differentiation and historical genetic connectivity; (2) whether spatial variation in temperature and precipitation is associated with genome‐wide patterns of genetic differentiation that imply local adaptation; and (3) how projected future climate change may reshape the adaptive landscape and expose different lineages to unequal levels of climate vulnerability. Genetic offset, which predicts the genomic change a population would require to track predicted future climate conditions, provides a mean to address this last question and to identify lineages at risk of maladaptation (Fitzpatrick and Keller 2015). By integrating population genomics with environmental association analyses and forward‐looking genetic offset projections, we further evaluate the efficacy of a recent evolutionary framework for identifying management‐relevant lineages and for guiding proactive conservation strategies under accelerating climate change.

## 2 Materials and Methods

### 2.1 Individual Collection, DNA Extraction, and ddRADseq Library Preparation

To cover the full geographic range of *E. formosana* , we sampled individuals from 26 localities spanning its known distribution (Figure 1D and Data S1). To evaluate how well these samples represent the species' overall range, we also compiled historical occurrence records from published studies and biodiversity databases (Hsu et al. 2021; Lin and Shih 2025; TaiBIF 2025; Yoshida et al. 2015; Yago et al. 2018, 2023). To reconstruct the evolutionary history of *E. formosana* , we included one congenic species, *E*. *thibetana*, as an outgroup, following the phylogenetic framework of Toussaint et al. (2020). In total, 80 individuals were analyzed, comprising 79 *E. formosana* and 1 *E*. *thibetana*. Detailed sampling information for all individuals is provided in Data S1.

Genomic DNA was extracted from a single leg of each specimen using the Gentra Puregene Tissue Kit (Gentra Systems) following the manufacturer's protocol. The ddRADseq library preparation followed established protocols (Peterson et al. 2012; De Vivo et al. 2023) with minor modifications. Approximately 200 ng of genomic DNA per individual was digested using the restriction enzymes EcoRI and MseI (New England Biolabs). Adapter sequences containing unique barcodes were ligated to the digested fragments using T4 ligase (New England Biolabs). Ligation products were purified individually using 1.8× AMPure XP magnetic beads (Beckman Coulter). Purified fragments were then amplified using an eight‐cycle PCR to incorporate Illumina sequencing adapters. PCR products were pooled in equimolar amounts, followed by an additional bead‐based purification step. All 80 samples were combined into a single sequencing library. Prior to sequencing, fragments between 300 and 800 bp were size‐selected using a Pippin Prep system (Sage Science). Single‐end 150‐bp sequencing was performed on an Illumina HiSeq 2500 platform at the NGS High Throughput Genomics Core, Biodiversity Research Center, Academia Sinica (Taiwan).

### 2.2 Data Processing, SNP Discovery and Filtering

Raw ddRADseq reads were processed using the STACKS v2.62 pipeline (Catchen et al. 2013; Rivera‐Colón and Catchen 2022). Demultiplexing was performed with *process_radtags* under default settings, retaining only reads containing an intact EcoRI cut site, a valid barcode, and no Illumina adapter contamination. Individuals with fewer than 50,000 retained reads were removed to ensure adequate sequencing depth. Two datasets were subsequently generated: a full dataset including all filtered *E. formosana* individuals together with the outgroup *E*. *thibetana*, hereafter denoted as Dataset A, and a species‐only dataset comprising only *E. formosana* individuals, hereafter denoted as Dataset B. Each dataset was assembled independently using the *denovo_map.pl* pipeline. To identify optimal parameter values for *M* (the number of mismatches allowed among stacks within individuals) and *n* (the number allowed when matching loci across individuals), we applied the r80 criterion (Mastretta‐Yanes et al. 2015; Paris et al. 2017), testing matched values of *M* and *n* from 1 to 8. Loci were retained only if present in at least 80% of individuals within each population, and the optimal parameter set was defined as the point at which the number of r80 loci plateaued, identified by examining the incremental change in retained loci between consecutive parameter combinations.

For both datasets, VCF files were generated using the STACKS *populations* module, with populations defined at the species level. For the Dataset A, loci were required to occur in at least 50% of individuals within the population, be present across all populations in the dataset, and have a minimum minor allele count of three (−min‐mac 3). For the Dataset B, the within‐population retention threshold was increased to 80%, while retaining the same criteria for presence across populations and minor allele count. Haplotype‐wise filtering (−filter‐haplotype‐wise) was enabled so that missing data thresholds were applied at the locus rather than the SNP level. For analyses sensitive to linkage and non‐neutrality, we excluded loci deviating significantly from Hardy–Weinberg equilibrium and retained only the first SNP per RAD locus (−write‐single‐snp) to minimize physical linkage. Ultimately, three specific VCF files were produced for downstream analysis: (1) Dataset A includes the outgroup for phylogenetic reconstruction. (2) Dataset B contains exclusively neutral loci and is utilized for population structure, gene flow, and conservation genetic indices. (3) Dataset B incorporates both neutral and non‐neutral loci for environmental genomic analyses, which benefit from denser SNP.

### 2.3 Population Genomic Structure

To characterize the genetic structure of *E. formosana* across Taiwan and evaluate the consistency of inferred population patterns across methods, we applied three complementary clustering approaches: ADMIXTURE (v1.3.0; Alexander et al. 2009), sNMF (Frichot et al. 2014), and snapclust (Jombart 2008). For ADMIXTURE, the VCF file was converted to PLINK format using PLINK v1.3.0 (Purcell et al. 2007), and structure was inferred across *K* = 1–8 using fivefold cross‐validation, with the optimal *K* selected as the value minimizing cross‐validation error. For sNMF, the VCF file was converted to geno format using the *vcf2lfmm* function in the LEA package (Frichot and François 2015), and structure was inferred using the *snmf* function over *K* = 1–8 with 10 replicates per value. The best‐supported lineage number was selected as the *K* yielding the lowest cross‐entropy score. sNMF provides ancestry estimates comparable to ADMIXTURE but with substantially shorter runtimes (Frichot et al. 2014). Snapclust analyses were performed by importing the VCF file into R with *read.vcfR* (Knaus and Grünwald 2017), converting them to genind objects via *vcfR2genind*, and estimating lineages using the *snapclust.choose.k* function in the adegenet package (Jombart 2008), with the optimal lineage number determined by both Akaike (AIC) and Bayesian (BIC) information criteria. Snapclust uses a likelihood‐based framework to identify the clustering configuration that best explains multilocus genotype variation, offering high accuracy and computational efficiency (Beugin et al. 2018).

### 2.4 Genomic Ordination and Spatial Genetic Correlation

To visualize the *E. formosana* lineage relationships inferred from the structure analyses, we performed principal component analysis (PCA). Prior to ordination, missing genotypes were replaced by mean allele frequencies across individuals using the *tab* function in adegenet package (freq = TRUE, NA.method = “mean”). Overall genotype missingness across the retained SNPs was 8.8%. PCA was then conducted on the resulting allele‐frequency matrix using the *dudi.pca* function in the ade4 package (Dray and Dufour 2007), with data centered and scaled (center = TRUE, scale = TRUE). The first two principal components (PC1 and PC2) were visualized using the *fviz_pca_ind* function in the *factoextra* package (Kassambara and Mundt 2020).

To assess the correspondence between genetic and geographic differentiation, we performed Procrustes analysis (Wang et al. 2012; Knowles et al. 2016) by comparing PC1 and PC2 with the geographic coordinates (latitude and longitude) of each individual. The analysis was implemented using the *protest* function in the vegan package (Dixon 2003), which identifies the optimal transformation minimizing the sum of squared Euclidean distances between the genetic and geographic configurations while preserving relative pairwise distances within each map. Statistical significance of the genetic–geographic association was evaluated using 10,000 permutations.

### 2.5 Phylogenetic Inference

We reconstructed the phylogenetic trees of *E. formosana* at both maximum likelihood and lineage approaches using IQ‐TREE 2 (Minh et al. 2020) and SVDquartets (Chifman and Kubatko 2014) respectively. Input files for both phylogenetic approaches were generated from the filtered VCF using *vcf2phylip.py* script (Ortiz 2019), retaining only loci present in more than half of the individuals, and converted into PHYLIP format for IQ‐TREE 2 and NEXUS format for SVDquartets. For the individual‐level phylogeny, model selection was conducted using MODELFINDER in IQ‐TREE 2 (Kalyaanamoorthy et al. 2017) under the Bayesian Information Criterion with ascertainment bias correction (+ASC), and branch support was assessed using 1000 ultrafast bootstrap replicates (Minh et al. 2013; Hoang et al. 2018) together with 1000 SH‐like approximate likelihood ratio test replicates (Guindon et al. 2010). To minimize potential inflation of ultrafast bootstrap support under model misspecification, we performed an additional step by enabling the –bnni option to further optimize ultrafast bootstrap trees by nearest neighbor interchange based directly on bootstrap alignments (Hoang et al. 2018). We additionally conducted 20 independent maximum‐likelihood searches to evaluate convergence and ensure topological consistency, selecting the tree with the highest log‐likelihood as the final topology. For the lineage‐level phylogeny, individuals were first assigned to lineages according to the results of population structure analyses, and the NEXUS files were analyzed in PAUP* (Swofford 2002) using the SVDquartets algorithm with 100 bootstrap replicates. All resulting phylogenetic trees were visualized in FigTree v1.4.4.

### 2.6 Gene Flow and Effective Migration Surfaces

We examined gene flow and potential barriers among populations using different analytical approaches. To estimate the strength and directionality of contemporary gene flow among lineages, as well as the inbreeding coefficient within each lineage, we used BA3‐SNPs (BayesAss v3.0.4; Mussmann et al. 2019), which infers both parameters under a Bayesian framework. Input files were generated by converting the filtered VCF into a genlight object using the *vcfR2genlight* function in the dartR.base package, and then formatting it with *gl2bayesAss* function while assigning individuals to populations according to the lineages inferred from the structure analyses. Preliminary exploratory runs were conducted to identify appropriate mixing parameters for migration rates (dM), allele frequencies (dA), and inbreeding coefficients (dF) using the built‐in autotune function. Full analyses were then performed for both three and four lineages (*K* = 3, 4; see Results section), using 200,000,000 MCMC iterations with a burn‐in of 20,000,000 and a sampling interval of 1000. Mixing parameters were set to dM = 0.1, dA = 0.3, and dF = 0.025 for *K* = 3, and to dM = 0.2, dA = 0.3, and dF = 0.035 for *K* = 4. Effective sample sizes for all estimated parameters were assessed using TRACER v1.7 (Rambaut et al. 2018). Migration rates and inbreeding coefficients were considered significant when their 95% confidence intervals (mean ±1.96 × standard deviation) did not overlap zero. Gene flow patterns among lineages were visualized using the *chordDiagram* function in the circlize package (Gu et al. 2014).

In addition, to evaluate the potential barriers among *E. formosana* populations, we inferred spatial patterns of historical connectivity using fast estimation of effective migration surfaces (FEEMS; Marcus et al. 2021). FEEMS estimates gene flow across a landscape as a spatially explicit graph, estimating effective migration rates along graph edges to identify regional barriers and corridors of accumulated gene flow. The approach uses a Gaussian Markov Random Field within a penalized likelihood framework, where smoothing parameters (lambda and lambda_q) control the degree of regularization. We evaluated the range of lambda values from 10^−3^ to 10^2^ and lambda_q values from 10^−1^ to 10 using cross‐validation and selected the parameter set that minimized cross‐validation error. Model fit was further assessed by comparing observed genetic distances with fitted distances under the optimal sets of lambda values, allowing us to evaluate how well the inferred migration surface captured node‐specific variance in connectivity across the landscape.

### 2.7 Genetic Diversity and Identity Disequilibrium

To quantify genetic diversity within and among *E. formosana* populations in Taiwan, we estimated multilocus heterozygosity (MLH; Coltman et al. 1999) using the *MLH* function in the inbreedR package (Stoffel et al. 2016). MLH values were summarized at both the individual and lineage levels under the two alternative lineages according to the population structure analyses (*K* = 3 and *K* = 4), and differences among lineages, as well as pairwise contrasts, were evaluated using Kruskal–Wallis tests (Kruskal and Wallis 1952). To further characterize the distribution and variance of inbreeding within lineages, we estimated identity disequilibrium (g_2_; David et al. 2007) using the *g2_snps* function in inbreedR (Hoffman et al. 2014; Stoffel et al. 2016). The g_2_ statistic quantifies covariance in heterozygosity across loci and provides an indicator of heterogeneity in inbreeding levels among individuals. Confidence intervals for g_2_ were obtained via 1000 bootstrap resamples across individuals, and statistical significance was assessed through 1000 permutations of multilocus genotypes to test the null hypothesis of no variation in inbreeding within each lineage. All these analyses provide an integrated assessment of genetic diversity and inbreeding structure across the major lineages of *E. formosana* .

### 2.8 Long‐Term Demographic History Reconstruction

To reconstruct the long‐term demographic histories of *E. formosana* , we employed Stairway Plot 2 (v2.2; Liu and Fu 2020), which infers historical changes in effective population size (*N*_*e*_) from the site frequency spectrum (SFS) without requiring a priori specification of demographic models. Demographic trajectories were estimated separately for each of the four lineages identified in the population structure analyses. Because Stairway Plot 2 requires the total number of sequenced sites (*L*), including both polymorphic and invariant positions, we generated four lineage‐specific VCF files using the populations module with the same filtering criteria described above, while retaining all sites (−vcf‐all). After calculating *L* for each lineage, we accounted for linkage among SNPs within the same RAD locus by using a customized Perl script (https://www.biostars.org/p/313701/) to randomly retain a single SNP per RAD locus for each lineage. Folded SFS input files were subsequently generated using easySFS (https://github.com/isaacovercast/easySFS), which converts VCF files into SFS while accommodating missing data. For each lineage, we selected the projection that maximized the number of segregating sites, following the recommendations of Gutenkunst et al. (2009). Finally, demographic histories were inferred assuming a mutation rate of 2.9 × 10^−9^ substitutions per site per generation, based on estimates from a nymphalid butterfly (Keightley et al. 2015), and a generation time of 1 year, consistent with the known life cycle of *E. formosana* (Hsu et al. 2021).

### 2.9 Environmental Genomic Associations and Climate Vulnerability

To identify associations between environmental and genomic lineages in *E. formosana* , we applied RDAforest (Matz and Black 2025), an extension of redundancy analysis that integrates the polygenic basis of adaptation with the flexibility of random forest regression. Unlike standard SNP‐by‐SNP association tests, this method evaluates multilocus covariance patterns and accommodates nonlinear, non‐monotonic, and interactive effects among predictors. RDAforest summarizes genome‐wide variation through a principal coordinate ordination of the among‐individual genetic distance matrix. The leading axes of this ordination, hereafter genomic principal component (gPC), were retained as the multivariate response describing genome‐wide covariance structure. The model was trained using nineteen bioclimatic variables at 30‐arc‐second resolution obtained from the WorldClim database (v2.1; Fick and Hijmans 2017). To mitigate potential confounding effects arising from the strong correlation between genetic and geographic structure observed in our dataset, we incorporated log‐transformed pairwise geographic distances into the ordination. Furthermore, we did not apply a separate variance inflation factor or correlation‐based filter to the bioclimatic predictors; instead, multicollinearity was handled within RDAforest through its *mtry*‐based predictor‐selection procedure (Matz and Black 2025). Specifically, predictor importance was evaluated across 50 jackknife replicates while increasing *mtry*, and only predictors whose importance reliably increased with *mtry* (in at least 70% of replicates; *prop.positive.cutoff* = 0.7) were retained, whereas predictors that consistently decreased were considered unstable and removed. Because a higher *mtry* forces correlated predictors to compete as split candidates, this criterion preferentially removes predictors whose apparent importance is driven by collinearity rather than by an independent association with genomic variation.

Moreover, to improve spatial interpretability and to reduce noise associated with continuous environmental gradients, we visualized the spatial patterns of environmental random forest predictions using clustering based on turnover curves. Clusters were subsequently merged at a level of 0.45, resulting in five environmental clusters that broadly correspond to vegetation zonation across mountainous regions of Taiwan (Li et al. 2013). Following clustering, environmental assignments for each individual were extracted from the grid cell corresponding to their specific GPS sampling location. The relationships between these assigned environmental clusters and the genomic lineages were then visualized using Sankey diagrams generated with the *sankeyNetwork* function in the networkD3 package (Allaire et al. 2017).

Finally, to evaluate how future climate change may reshape environmentally associated genomic patterns, we quantified genetic offset across the geographic range of *E. formosana* . Genomic composition was predicted for both present‐day and future climates using *ordinationJackknife* with 40 replicates, retaining the six leading gPC axes. Future climates were represented by 30‐arc‐second bioclimatic layers from the BCC‐CSM2‐MR climate model (Wu et al. 2019) for four future time periods (2021–2040, 2041–2060, 2061–2080, and 2081–2100) under four Shared Socioeconomic Pathway scenarios (SSP126, SSP245, SSP370, and SSP585), which represent a gradient from mild to severe climatic change (SSP 126 < SSP 245 < SSP 370 < SSP 585). Genetic offset was defined as the Euclidean distance between present‐day and future RDAforest‐predicted gPC values at each point on the landscape; that is, the distance metrics represent population‐specific vulnerability to future climate conditions. Because random forest regression cannot extrapolate beyond the environmental range represented in the training data (Matz and Black 2025), future grid cells whose projected conditions fell outside the calibration domain were not retained in the predictions and were classified as non‐analog. We did not assign genetic offset values to these cells because predictions there would be bound by the most extreme training values and would understate rather than quantify the true degree of environmental novelty (Fitzpatrick and Hargrove 2009). Non‐analog exposure was instead shown as a categorical indicator. We used these projections to quantify spatial variation in genetic offset and identify regions or lineages expected to experience the greatest shifts under future climates.

## 3 Results

### 3.1 Summary of Molecular Data

A total of 80,443,256 raw reads were generated from the ddRADseq library, of which 79,995,378 passed quality filtering. After removing individuals with insufficient retained reads (< 50,000), 70 individuals, comprising 69 *E. formosana* and one *E*. *thibetana*, were retained for downstream analyses, with an average of 1,141,557 high‐quality reads per sample (standard error = 138,990).

Using the r80 criterion, the optimal parameter combinations for the de novo assembly were M1n1 for the Dataset A and M2n2 for the Dataset B. Detailed information on the assembled loci for all r80 parameter sets is provided in Table S1. For analyses requiring unlinked and putatively neutral loci, the final datasets contained 518 SNPs for the Dataset A and 4529 SNPs for the Dataset B. For environmental genomic analyses, a total of 9269 SNPs, including both neutral and non‐neutral loci, were retained for the Dataset B after filtering.

### 3.2 Population Genomic Structure and Spatial Genetic Patterns

**FIGURE 2:**
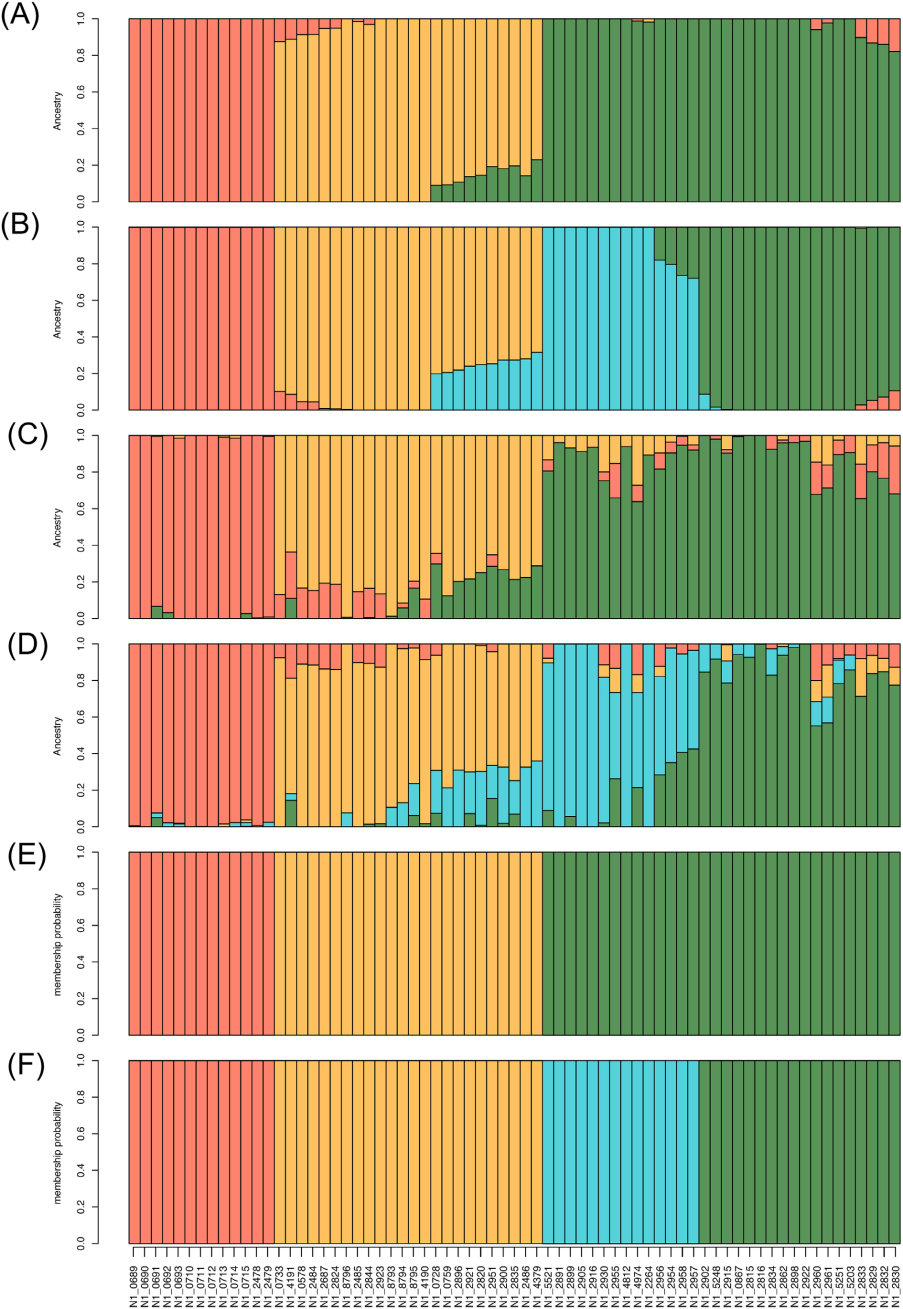
Genetic structure *of Euthalia formosana* inferred from three complementary clustering approaches. (A, B) Individual ancestry coefficients for *K* = 3 and *K* = 4 inferred using ADMIXTURE. (C, D) Individual ancestry coefficients for *K* = 3 and *K* = 4 inferred using sNMF. (E, F) Membership probability for *K* = 3 and *K* = 4 inferred using snapclust. Each vertical bar represents an individual, and colors denote estimated membership probabilities. Sample labels are displayed below each bar. Within each panel, individuals are arranged first by lineages, in the order southwest, northwest, northeast, and southeast, and then by their ancestry coefficient (structure score) within each lineage.

All three clustering approaches recovered broadly concordant population structure of *E. formosana* (Figure 2). Although the optimal number of lineages varied slightly among methods, ADMIXTURE supported *K* = 3, sNMF supported *K* = 4, and snapclust identified *K* = 4 under AIC but *K* = 1 under BIC. Both *K* = 3 and *K* = 4 consistently emerged as the best‐ or second‐best‐supported configurations (Figure S1). The agreement among these independent analytical frameworks indicates that the major features of population structure are robust and not driven by any single method. Under the *K* = 3 configuration, individuals were primarily separated into eastern, northwestern, and southwestern lineages (Figure 2A,C,E). Under *K* = 4, the eastern lineage was further subdivided into northeastern and southeastern lineages (Figure 2B,D,F). Although ancestry proportions differed slightly among ADMIXTURE, sNMF, and snapclust, the geographic distribution of lineages and individual assignments was highly consistent, with only minor differences in the proportions. This strengthens the biological validity of the inferred population structure. Furthermore, since the snapclust results provide unambiguous individual assignment, lineage assignments were based on these results for subsequent analyses.

PCA further supported these results. PC1 (7.3% of total variance) captured a pronounced separation between eastern and western individuals, consistent with the CMR acting as a major barrier to gene flow (Figure S2). PC2 (4.4%) primarily differentiated individuals within western Taiwan, mirroring the finer‐scale structure detected by the clustering analyses. Notably, western populations showed a broader spread along both principal components, whereas eastern populations formed a comparatively tight cluster. This pattern indicates more pronounced genomic subdivision in western Taiwan. Procrustes analysis confirmed a strong correspondence between genetic and geographic structure (*p* < 0.00001), with the dominant signal again reflecting the east–west partition imposed by the CMR (Figure S2B,D). However, the spatial distribution of lineages also reveals clear regional asymmetry: western lineages exhibit greater internal genetic subdivision than the eastern lineages. These results identify a consistent genetic structure between populations on opposite sides of the CMR.

### 3.3 Evolutionary Relationships and Contemporary Connectivity

**FIGURE 3:**
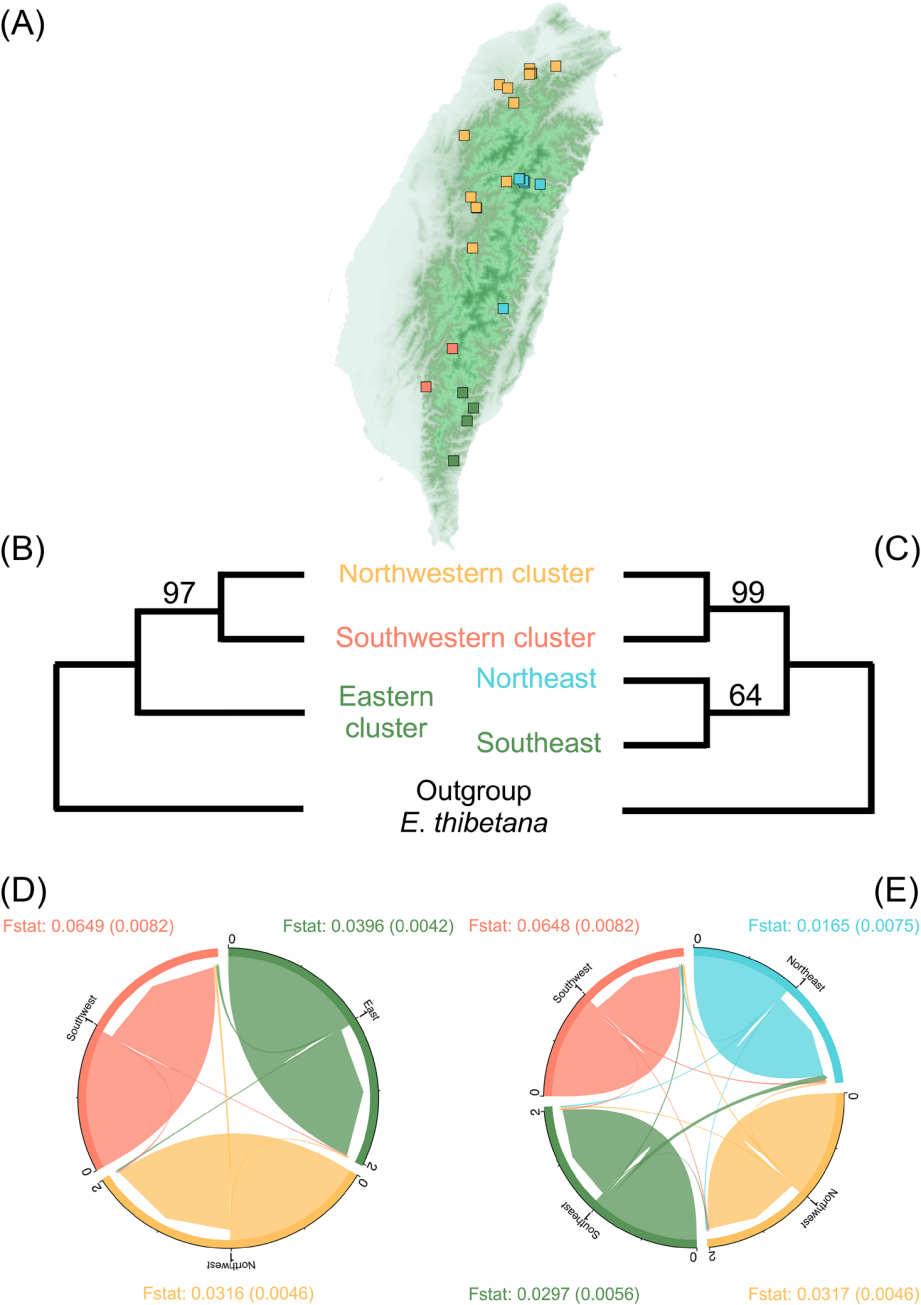
Species trees, contemporary gene flow, and within‐lineage inbreeding inferred under alternative genetic clustering schemes. (A) Geographic distribution of samples assigned to four lineages based on population structure analyses. (B) Species tree reconstructed using populations defined under the three‐lineage (*K* = 3) configuration. (C) Species tree reconstructed using populations defined under the four‐lineage (*K* = 4) configuration. Node support values in (B) and (C) represent bootstrap values based on 100 replicates. (D) Estimated contemporary migration rates among lineages and within‐lineage inbreeding coefficients under the three‐lineage configuration. (E) Estimated contemporary migration rates among lineages and within‐lineage inbreeding coefficients under the four‐lineage configuration. Across all panels, colors indicate regional lineages: Southwest (red), northwest (yellow), southeast (green), and northeast (blue). Under the *K* = 3 configuration, the southeast and northeast groups merge into a single eastern lineage.

The individual‐level phylogeny recovered a broadly ordered sequence of divergence among the major lineages of *E. formosana* , although support for many internal branches was relatively low (bootstrap < 70; Figure S3). Individuals from eastern Taiwan formed the earliest diverging assemblage, but northeastern and southeastern samples were intermingled rather than forming distinct monophyletic lineages. Northwestern individuals separated next, followed by the southwestern individuals, which was the only lineage to form a well‐supported monophyletic clade near the terminal end of the tree. This pattern indicates a spatially ordered sequence of divergence around the island, progressing from eastern to northwestern and then to southwestern regions. Given the limited resolution of some internal nodes, we interpret this phylogeny as reflecting broad spatial structure rather than a precise reconstruction of branching order. In contrast, the lineage‐level phylogeny placed the root between eastern and western groups under both the three‐ and four‐lineage configurations (Figure 3B,C), emphasizing the dominant role of the CMR in shaping deep genetic structure. Although root placement differed between ML‐ and lineage‐based trees, both phylogenetic relationships consistently revealed pronounced asymmetry in within‐region differentiation. Northeastern and southeastern individuals showed substantial intermixing, whereas the western lineages exhibited stronger support for a north–south split (bootstrap = 99) than the eastern lineages (bootstrap = 64), indicating more pronounced lineage differentiation within western Taiwan.

Bayesian estimates of recent migration indicated generally low levels of contemporary gene flow among major lineages, particularly under the three‐lineage configuration (Figure 3D,E). Average migration rates were 0.0142 (range = 0.0095–0.0209) for *K* = 3 and 0.0193 (range = 0.0119–0.0545) for *K* = 4. Under the four‐lineage model, the strongest asymmetry was detected between northeastern and southeastern lineages, with migration from the southeastern into the northeastern lineage (0.0545) exceeding the reverse direction (0.0152). This southeast‐to‐northeast movement represented the highest among‐lineage gene flow detected across all populations.

To further characterize spatial variation in connectivity, we inferred effective migration surfaces using FEEMS. The optimal parameter combination (lambda = 0.17; lambda_q = 10) minimized cross‐validation error (Figure S4B: model b) and produced a high overall model fit (*R* ^2^ = 0.922; Figure S4C). The resulting surface identified the CMR as the dominant barrier to movement, with stronger restrictions in the southern portion of the island than in the north. In addition, consistently low effective migration values were detected around the southwestern lineage, indicating pronounced isolation relative to other populations (Figure S4A: model b).

### 3.4 Genetic Diversity and Inbreeding Levels Across Lineages

**FIGURE 4:**
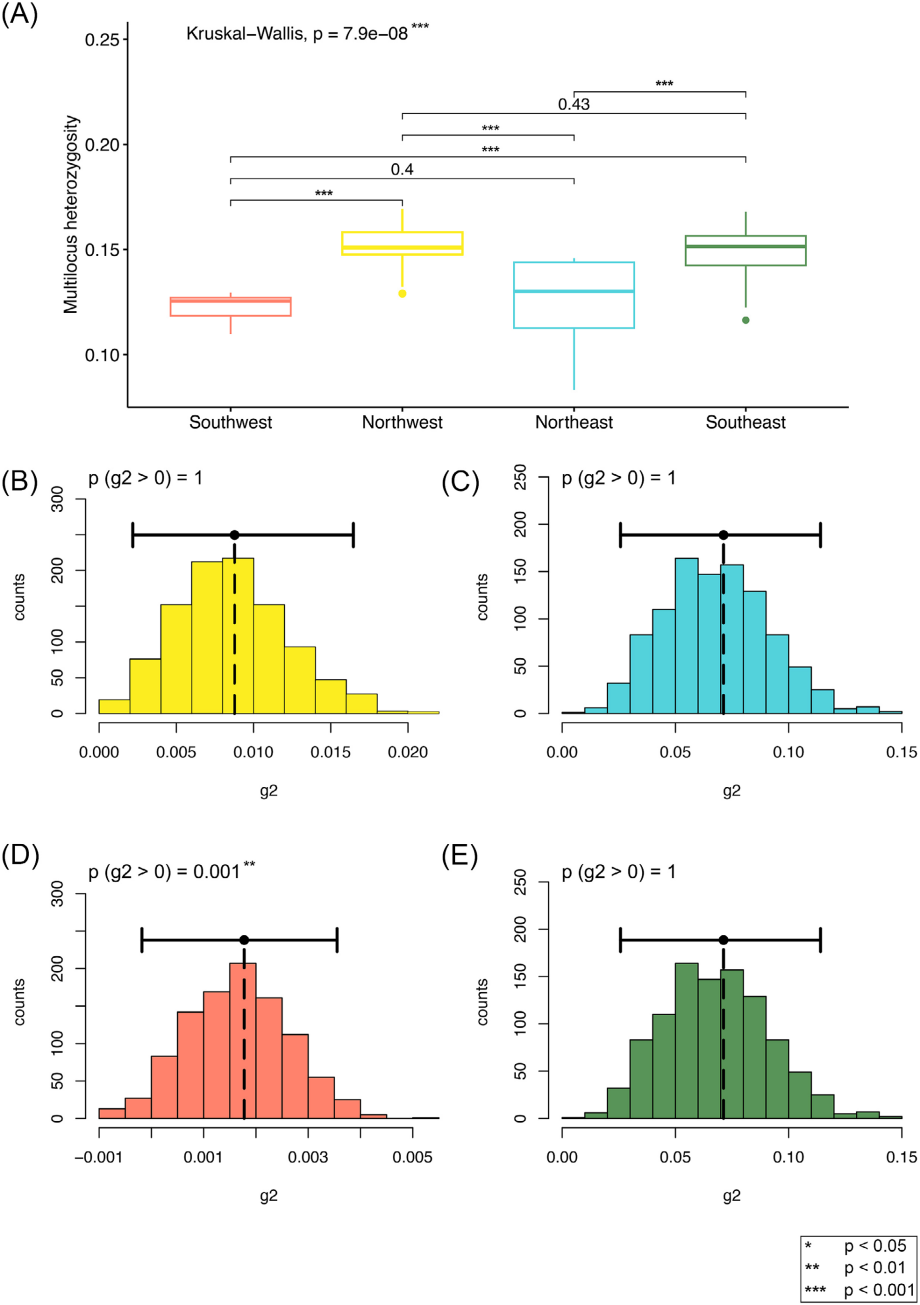
Genetic diversity and identity disequilibrium (g_2_) of *Euthalia formosana* in Taiwan. (A) Boxplots showing the distribution of multilocus heterozygosity (MLH) among individuals grouped into four lineages (*K* = 4). Differences in MLH among lineages were assessed using Kruskal–Wallis tests. (B–E) Distributions of the g_2_ statistic for the northwest (B), northeast (C), southwest (D), and southeast (E) lineages, estimated from 1000 permutations. Across all panels, colors indicate regional lineages: Southwest (red), northwest (yellow), southeast (green), and northeast (blue). Asterisks indicate significance levels: * means *p* < 0.05, ** means *p* < 0.01, and *** means *p* < 0.001.

Genetic diversity, quantified by MLH, differed significantly among *E. formosana* lineages under the four‐lineage configuration (*p* = 7.9 × 10^−8^; Figure 4A). The southwestern lineage exhibited the lowest diversity (mean MLH = 0.123), which was significantly lower than the values observed in the northwestern (mean MLH = 0.151) and southeastern lineages (mean MLH = 0.148). However, no significant difference in MLH was observed between the southwestern and northeastern lineages (mean MLH = 0.125). Furthermore, no significant differences were detected among the northwestern, northeastern, and southeastern lineages under this configuration. Individual‐level MLH values are provided in Figure S5.

To assess inbreeding patterns, we first estimated identity disequilibrium (g_2_) under the four‐lineage configuration. Among all lineages, only the southwestern lineage exhibited a significant g_2_ value, indicating detectable variance in individual inbreeding levels within this region (Figure 4B–E). Bayesian estimates of the inbreeding coefficient (*F*stat) further supported this pattern: the southwestern lineage showed the highest mean *F*stat, approximately four times greater than that of the northeastern lineage, which exhibited the lowest inbreeding levels (Figure 3E). These results consistently identify the southwestern lineage as genetically depauperate, highly inbred, and strongly isolated relative to other populations.

### 3.5 Long‐Term Demographic History

Stairway Plot analyses revealed broadly similar long‐term demographic trajectories across the four lineages of *E. formosana* (Figure S6). All lineages exhibited relatively stable effective population sizes during the early to mid‐Holocene, followed by two periods of population decline during the late Holocene. The first and most pronounced reduction in effective population size occurred approximately 2.0–4.5 kya (Figure S6a), with particularly strong declines inferred for the northwestern and southeastern lineages. A second, more recent decline was detected around 0.2–0.45 kya (Figure S6b), although this signal was weaker and more variable among lineages. Overall, the four lineages shared a largely concordant demographic history characterized by long‐term stability followed by late‐Holocene declines, with only modest differences in timing and magnitude among regions. Differences in effective population size were consistent with the previous described patterns of genetic diversity and inbreeding, with the more genetically diverse and less inbred lineages maintaining larger long‐term effective population sizes.

### 3.6 Environmental Genomic Associations and Climate Vulnerability of the Formosan Duke Across Taiwan

**FIGURE 5:**
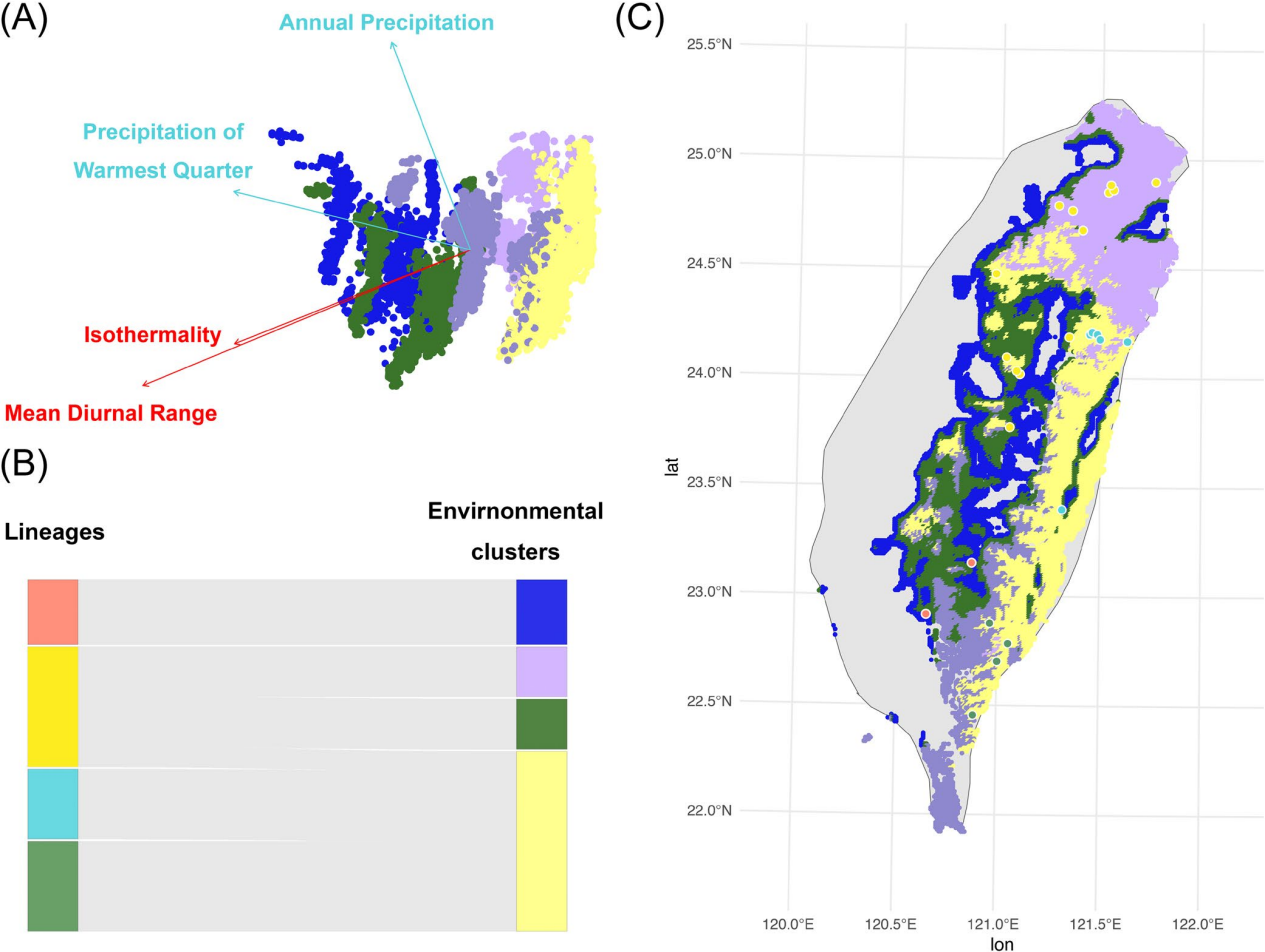
Environmental genomic associations in *Euthalia formosana* inferred from *RDAforest* analyses. (A) PCA based on Random Forest predictions across the landscape, with clustering based on genomic–environmental turnover curves. The four most influential bioclimatic variables retained after the *mtry*‐based filtering procedure are projected onto the PCA space, with arrows indicating the direction and strength of their associations. (B) Relationships between the lineages of *E. formosana* and the environmental clusters, as defined by turnover‐curve clustering across Taiwan. Lineages are colored as follow: southwest (red), northwest (yellow), southeast (green), and northeast (blue). (C) Spatial projection of turnover‐curve clustering across Taiwan, illustrating geographic patterns of genomic–environmental turnover.

Four bioclimatic variables were retained in the RDAforest analysis after applying the *mtry*‐based filtering procedure to account for inflated importance scores due to correlated predictors, namely mean diurnal range (bio_2), isothermality (bio_3), annual precipitation (bio_12), and precipitation of the warmest quarter (bio_18). In total, these variables explained 32.94% of the total genomic variance captured by the model (Figure S7A). Using these predictors, we identified five environmental clusters based on turnover patterns in genomic–environmental associations. A PCA of RDAforest‐predicted environmental values illustrated how these clusters were distributed along the major axes of multivariate climatic variation (Figure 5A), and their spatial projections revealed clear geographic structuring across Taiwan (Figure 5C). A dendrogram summarizing relationships among environmental clusters further grouped them into two higher‐level climatic assemblages (Figure S7B), which broadly corresponded to northern–eastern versus mid‐ to southwestern regions of the island.

Although partial overlap existed between genetic lineages and environmental clusters (Figure 5B), most lineages of *E. formosana* occupied relatively restricted portions of climatic space. Notably, the southwestern lineage was unique in being confined to a single environmental cluster (dark blue environmental cluster; Figure 5B), which was strongly associated with precipitation of the warmest quarter (Figure 5A). In contrast, the northwestern lineage was associated with multiple environmental clusters, suggesting broader climatic occupancy. The two eastern lineages, northeastern and southeastern, were both confined to the same environmental cluster (yellow environmental cluster; Figure 5B), which was positioned at the opposite end of the climatic gradient from the southwestern lineage. These results indicate that most lineages of *E. formosana* are climatically constrained, with the southwestern lineage showing the narrowest realized climatic niche.

**FIGURE 6:**
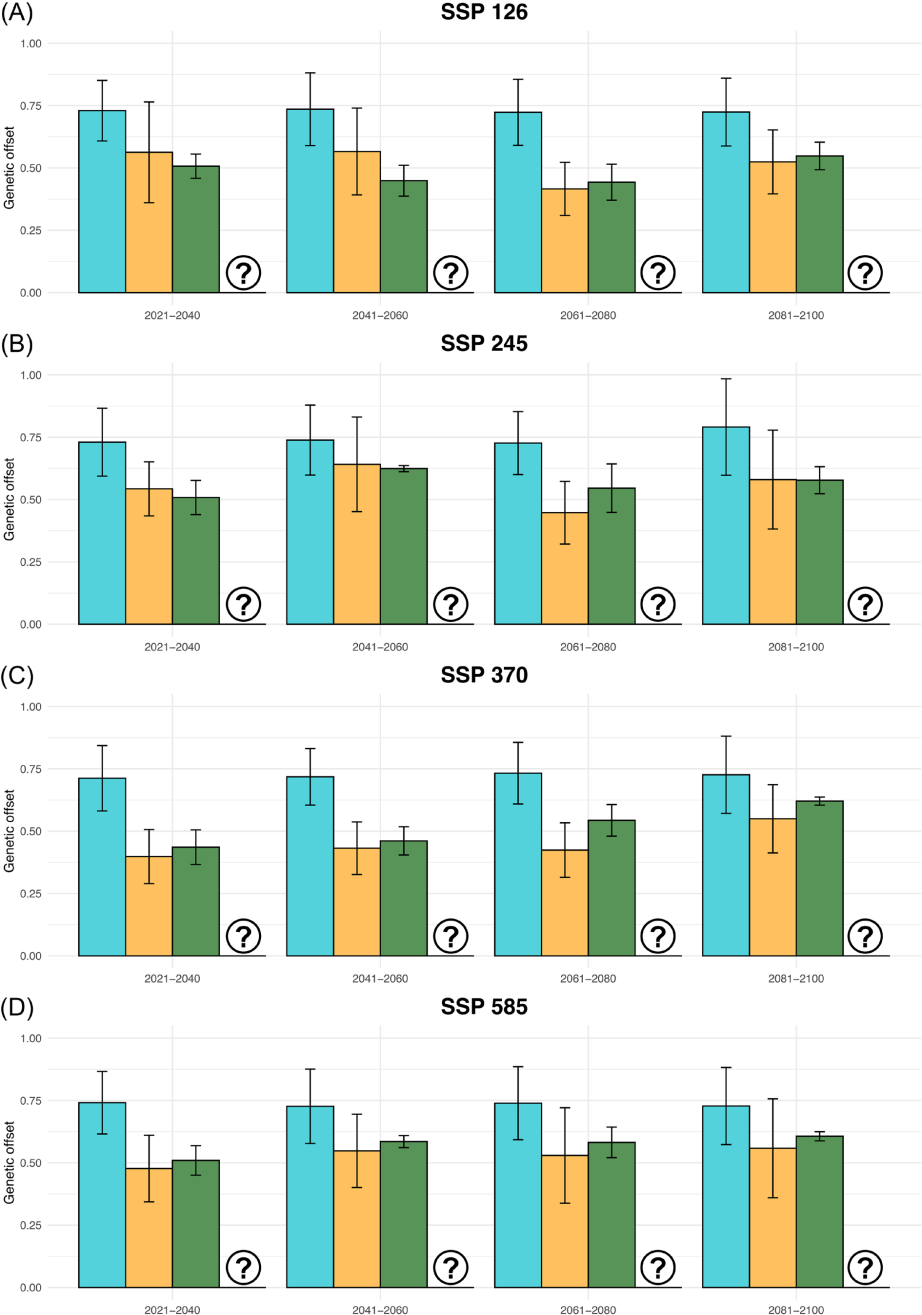
Genetic offset projected under future climate scenarios for the four lineages of *Euthalia formosana*. Predicted genetic offset across four future time periods (2021–2040, 2041–2060, 2061–2080, and 2081–2100) under (A) SSP126, (B) SSP245, (C) SSP370, and (D) SSP585. Colors denote regional lineages: southwest (red), northwest (yellow), southeast (green), and northeast (blue). Genetic offset for the southwest lineage could not be estimated for any time period or SSP scenario (indicated by a question mark) because projected future climatic conditions fall outside the environmental domain used to calibrate the model, precluding reliable extrapolation. Non‐analog conditions indicate the absence of a present‐day environmental equivalent, suggesting environmental change beyond the range represented by the estimated genetic offset values rather than a direct prediction of demographic extinction.

**FIGURE 7:**
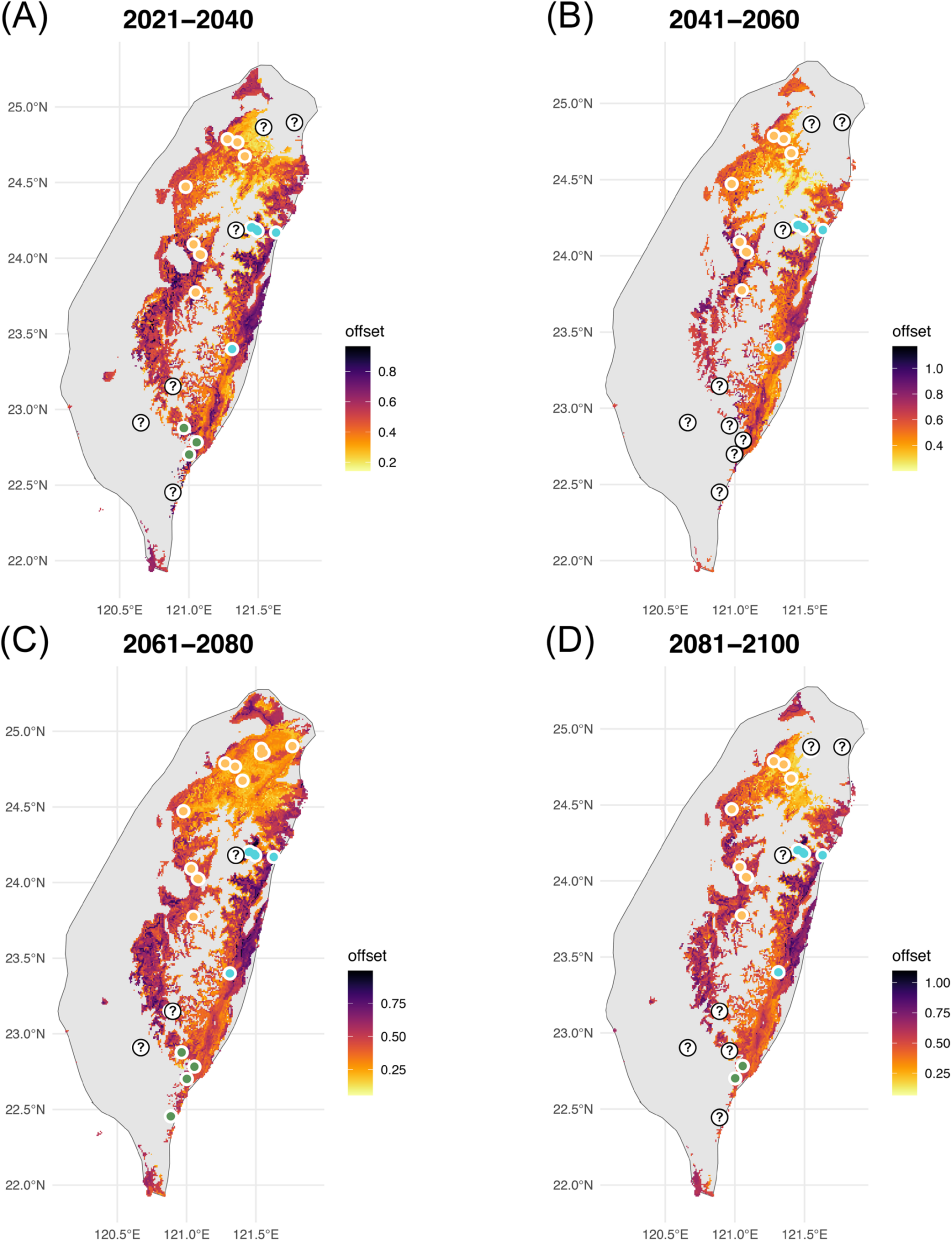
Spatial projection of genetic offset across Taiwan under SSP245 for the four lineages of *Euthalia formosana*. Panels show predicted offset for (A) 2021–2040, (B) 2041–2060, (C) 2061–2080, and (D) 2081–2100. Darker colors indicate greater predicted genomic change required for populations to remain environmentally matched. Point colors denote regional lineages: Southwest (red), northwest (yellow), southeast (green), and northeast (blue). Populations whose projected future conditions fall outside the calibration domain are marked as non‐analog (offset not estimated; shown as a question mark). Non‐analog conditions in southwestern Taiwan were consistent across all four SSP scenarios, including the low‐emissions SSP126 pathway (Figures S8–S10).

Genetic offset projections revealed pronounced spatial and lineage‐specific heterogeneity across Taiwan under all future climate scenarios (Figures 6, 7, and S8–S10). The spatial distribution of offset under the intermediate SSP245 scenario is shown in Figure 7, with the remaining scenarios provided in Figures S8–S10. For the southwestern lineage, projected future climatic conditions consistently fell outside the environmental domain used to calibrate the model across all four SSP scenarios and time periods, precluding the assignment of quantitative offset values and indicating persistent exposure to non‐analog future climates (i.e., projected conditions with no equivalent present‐day range of environmental conditions used to calibrate the model) in this region. Among the remaining lineages, the northeastern lineage consistently exhibited the highest genetic offset values across all scenarios and future periods, suggesting that the largest magnitude of environmentally associated genomic change would be required for populations in this region to remain climatically matched. In contrast, the northwestern and southeastern lineages showed comparatively lower offset values, with only modest increases toward later time periods under low to intermediate (SSP126 and SSP245) and high‐emissions (SSP370 and SSP585) scenarios. However, population‐level projections revealed that exposure to non‐analog future climates was not restricted to the southwestern lineage: several populations within the southeastern lineage, as well as north populations and a high‐elevation population (Shangguanyuan) from the northwestern lineage, repeatedly fell outside the future environmental domain across all scenarios (Figures 7 and S8–S10).

## 4 Discussion

Our findings show that the interplay of topographic structure, climatic heterogeneity, and dynamic historical connectivity in Taiwan has provided sufficient opportunity for the emergence of distinct lineages. Specifically, we found notable population genetic asymmetry in the genetic structure of the Formosan Duke that defies simple phylogeographic explanations. While the CMR stands as the undeniable barrier of the species' primary east–west divergence, similar to the cases observed across a broad spectrum of Taiwanese biota (Wang et al. 2007; Huang and Lin 2010, 2011; Lin et al. 2008, 2023), our results highlight a pronounced contrast in how subsequent population subdivision has unfolded on either side of this barrier. Whereas eastern lineages retain signatures of genetic continuity, western populations exhibit profound genetic subdivision and low contemporary connectivity, mirroring regional contrasts in land‐use intensity and habitat fragmentation (Chen et al. 2019; Liu 2019). Specifically, the western part of Taiwan has been historically as well as currently more heavily developed than the eastern part of Taiwan. Furthermore, although long‐term demographic reconstructions point to broadly shared post‐Holocene histories across regional lineages, our results reveal that the southwestern lineage is confined to a narrow climatic range and has experienced geographic isolation. As a result, this southwestern lineage highlights a critical empirical example that illustrates a shift from historical local specialization to specific environmental conditions, toward modern, and potentially future, vulnerability to anthropogenic disturbance and climate change. More broadly, this phenomenon has inadvertently set the stage for uneven adaptive potential, leaving specific populations disproportionately vulnerable to a rapidly shifting climate. In the sections that follow, we first discuss how tracing the history of these evolutionarily distinct lineages reveals their associations with the topographic complexity of Taiwan. We then examine how current climatic heterogeneity and varying levels of anthropogenic disturbance continue to drive the contrasting pattern of genetic subdivision between eastern and western Taiwan, leading to different lineage‐specific genetic statuses and vulnerabilities. Ultimately, we propose that incorporating these historical legacies alongside contemporary genetic patterns establishes a robust framework for devising conservation strategies that protect the evolutionary potential and future adaptive capacity of this endemic butterfly in the context of global climate change.

### 4.1 Phylogeographic History and Within‐Island Diversification

We found that the current population structure of the Formosan Duke is associated with long‐term phylogeographic processes across Taiwan's complex topography. Across multiple analytical approaches, we detected a predominant east–west genetic divide associated with the CMR. This indicates that the CMR is a persistent barrier to gene flow for the lowland species. This pattern echoes those reported for a wide range of Taiwanese taxa, including plants (Lin et al. 2023), amphibians (Lin et al. 2012), reptiles (Lin et al. 2008), fishes (Wang et al. 2007), and insects (Huang and Lin 2010, 2011). Additionally, our phylogenetic analyses provide clear evidence of within‐island diversification. All the Formosan Duke lineages form a monophyletic group and share a recent common ancestor (Figure S3). Similar patterns of population structure within Taiwan have been reported in other endemic taxa (Lin et al. 2025; Yeh et al. 2025). Notable, our individual‐level phylogeny reveals a novel counterclockwise pattern of diversification, rather than reciprocal diversification between the two sides of the CMR (Figure 3). Specifically, lineages from eastern Taiwan diverged first, followed by divergence of the northwestern lineages, and subsequently the southwestern lineages. This sequential diversification pattern may reflect a distinctive phylogeographic history of Taiwan as a continental island (Wang et al. 2022). Ancestral lineages are thought to have originated primarily from the adjacent mainland to the west (i.e., mainland China; He et al. 2018; Ali and Fritz 2025). However, the low bootstrap values on numerous internal branches may also indicate phylogenetic uncertainty associated with incomplete lineage sorting. Ancestral polymorphisms often fail to sort neatly into lineages, and this phenomenon has been documented to result in erroneous root placement in individual‐level phylogeny (Shen et al. 2017). Nevertheless, despite these uncertainties, our results demonstrate that different evolutionary trajectories can still present within a single island system, even among endemics diversifying within Taiwan.

### 4.2 Asymmetric Diversification and Lineage‐Specific Genetic Status

Beyond the primary east–west split, our results further show that genetic differentiation across populations between the two sides of the barrier is not spatially symmetric. This is supported by the PCA (Figure S2A,C), Procrustes analyses (Figure S2B,D), contemporary gene flow (Figure 3E), phylogenies (Figures 3B,C and S3), and effective migration surfaces (Figure S4). While eastern lineages showed weak subdivision, western lineages exhibited pronounced north–south subdivision. Similar east–west asymmetries have been observed in other Taiwanese endemics (e.g., Wang et al. 2022). However, the magnitude of subdivision in the Formosan Duke is more apparent. This contrast between east and west suggests that post‐divergence processes have differed on either side of the CMR. Our genotype–environment association analyses further suggest that climatic heterogeneity has contributed to present‐day genetic differentiation, as regional lineages are sorted into distinct environmental clusters (Figure 5). Results from the RDAforest analysis identified five distinct environmental clusters that correspond to previously identified mountainous vegetation zones in Taiwan (Figure 5C; Li et al. 2013). The southwestern lineage of the Formosan Duke is uniquely restricted to foothill and submontane evergreen forests correlated with summer monsoon rainfall. In contrast, the northwestern lineage shows broader climatic occupancy across multiple environmental clusters, whereas eastern lineages are confined to stable, moisture‐persistent montane evergreen and mixed cloud forests. These patterns indicate that most lineages occupy narrow environmental niches. Only the northwestern population shows environmental flexibility (Figure 5). Late‐Holocene climatic fluctuations (De Vivo et al. 2023) and spatially heterogeneous land‐use change likely modified landscape connectivity. Urban development has been more intense in western Taiwan than in the east (Chen et al. 2019; Liu 2019). These factors could intensify the north–south subdivision in the west. Such processes plausibly contributed to asymmetric diversification between eastern and western lineages and have direct consequences for lineage‐specific genetic status and vulnerability. The combination of limited connectivity and environmental breadth has likely triggered recent population declines. The combined impact is most evident in the southwestern lineage. It shows reduced multilocus heterozygosity (Figure 4A), pronounced identity disequilibrium (Figure 4D), and elevated inbreeding coefficients (Figure 3D,E).

### 4.3 Predicted Maladaptation and Lineage‐Specific Climate Vulnerability

Our findings further suggest that the evolutionary processes driving within‐island differentiation may also result in heterogeneous vulnerability to future climate change. While adaptation along environmental gradients facilitates persistence under specific local conditions, it can simultaneously reduce a lineage's niche breadth, thereby limiting its capacity to respond to novel or rapidly shifting environments. This evolutionary trade‐off is most pronounced in the southwestern lineage of the Formosan Duke, which exhibits a narrow climatic occupancy, low genetic diversity, and elevated inbreeding. Such factors limit the availability of standing genetic variation and hinder the acquisition of adaptive variants through gene flow, and thus constrain the lineage's ability to track environmental shifts (Nagel et al. 2017; Park and Talbot 2018; Williams and Dumroese 2013; Pedlar et al. 2012). This expectation is consistent with our genetic offset projections, which highlight both potential mismatch and, in some regions, non‐analog conditions that increase uncertainty. For the southwestern lineage, genetic offset projections consistently fell outside the environmental domain used to calibrate the model across all four SSP scenarios (SSP126, SSP245, SSP370, and SSP585) and time periods. This finding indicates that the assignment of quantitative offset values was not feasible and suggested the possibility of exposure to future non‐analog climates. The absence of an offset estimate is informative in itself. It suggests that future conditions may not have any contemporary analogue, and that present‐day genotype–environment relationships may not transfer to predict future association (Fitzpatrick and Hargrove 2009; Beale and Lennon 2012). Non‐analog projections do not directly imply habitat unsuitability. Nevertheless, these findings do highlight considerable uncertainty and the possibility of substantial mismatch (Fitzpatrick and Hargrove 2009). Importantly, non‐analog exposure was also detected in several populations in southeastern lineage and in isolated northern and high‐elevation populations within the northwestern lineage (Figures 7 and S8–S10). This raises concern for the southwestern lineage, as low genetic diversity, elevated inbreeding, and limited connectivity may limit adaptive responses and demographic resilience. Among the remaining lineages, the northeastern lineage showed the highest offset values, implying that large environmentally associated genomic changes would be required to remain climatically matched. In contrast, the northwestern and southeastern lineages showed lower offsets.

### 4.4 Management Implications and Future Directions

Given the single‐colonization history, limited gene flow, and pronounced lineage differentiation of the Formosan Duke populations, conservation efforts should prioritize the maintenance of both local genetic diversity and adaptive potential. Habitat restoration and genetically informed management of connectivity among populations belonging to the same evolutionary lineage may facilitate the exchange of novel genetic variants, thereby buffering small and isolated populations, particularly the southwestern lineage, from further genetic erosion while preserving their evolutionary distinctiveness (Pimm et al. 2006; Johnson et al. 2010; Aguilar‐Gómez et al. 2025). However, such interventions may also come with potential risks, as increased gene flow may introduce maladaptive alleles that reduce fitness in highly specialized populations (Frankham et al. 2011; Côte et al. 2014; Waller 2015). Careful evaluation of the genetic composition of source and sink populations is therefore essential prior to any translocation or assisted gene flow management. Genetic rescue through controlled translocation may represent a proactive strategy for enhancing local genetic diversity (Chen et al. 2022; White et al. 2023; Łabiszak and Wachowiak 2025), as suggested by recent efforts in the Formosan salmon (Lee et al. 2025), but such approaches must be implemented cautiously to avoid disrupting local adaptations or homogenizing evolutionarily independent lineages, as illustrated by the unfortunate case of the Chinese giant salamander (Yan et al. 2018). The Formosan Duke also highlights the increasing vulnerability of organisms inhabiting lower‐montane evergreen forests, which is one of Taiwan's long‐standing frontiers of human–nature conflict (Hsieh et al. 1996). Although the Formosan Duke is currently listed as nationally near threatened (NNT) in the updated Red List of Butterflies of Taiwan (Wei et al. 2025), its endemic status and well‐resolved diversification history make it an ideal species for engaging local communities and the general public. Specifically, it may become an excellent candidate for an evidence‐based biodiversity restoration program that involves local stakeholder engagement, including, but not limited to, school teachers and students and organized butterfly conservation groups.

The developing of a high‐quality chromosome‐level reference genome for this butterfly would represent a critical next step toward informing the conservation of the species. With the reference genome, signatures of recent anthropogenic impact could be detected at a finer temporal scale (e.g., Shen et al. 2025), and loci under selection across lineages could be identified and annotated, including their potential functions, particularly in the southwestern lineage. Additionally, a finer scale geographical sampling, especially at the margins of the southwestern lineage and in the non‐analog regions, would help evaluate connectivity and detect contemporary gene flow among neighboring populations of the same lineage. Finally, if new evidence indicates that the isolation and demographic decline of the southwestern lineage stem from anthropogenic deforestation of lower‐montane evergreen forests, restoring these forests in southwestern Taiwan would become a conservation priority to reconnect fragmented populations within the lineage. Habitat‐based restoration offers a cautious management strategy by re‐establishing natural connectivity among populations that share a common evolutionary history rather than introducing variants from distinct lineages. This approach minimizes the potential risks of genetic admixture while supporting the long‐term persistence of this most vulnerable lineage.

## 5 Conclusions

This study highlights the complex consequences of within‐island diversification in island endemics and illustrates how genomic analyses can help elucidate the evolutionary processes and potential lineage‐specific vulnerability within a single species. Our findings also provide a genomic perspective that can inform biodiversity conservation under rapid climate change. In the Formosan Duke, the southwestern lineage may face elevated risk associated with ongoing habitat loss and future climatic conditions. Similar dynamics may also affect other taxa restricted to southwestern Taiwan. More broadly, our framework provides a basis for evaluating persistence and vulnerability in other island endemics and supports the development of evidence‐based conservation strategies based on population genomic data.

## Conflicts of Interest

The authors declare no conflicts of interest.

## Supporting information


**Data S1:** Sampling information and genetic cluster assignments inferred by snapclust. Assignments are shown for *K* = 3 and *K* = 4.
**Figure S1:** Selection of the optimal number of lineages (K = 1–8) across population structure analyses. (A) Cross‐validation error for ADMIXTURE clustering solutions. (B) Cross‐entropy values for clustering solutions inferred using sNMF. (C) AIC values for clustering solutions inferred using snapclust. Together, these metrics support selection of the most appropriate number of lineages for downstream analyses.
**Figure S2:** Geographic–genetic correspondence in Euthalia formosana across Taiwan. (A) PCA of individuals grouped under the K = 3 solution. (B) Procrustes comparison of geographic locations (squares) and genetic configurations (circles) under K = 3. Colors: southwest (red), northwest (yellow), east (green). (C) PCA of individuals grouped under the K = 4 solution. (D) Procrustes comparison under K = 4. Colors: southwest (red), northwest (yellow), southeast (green), northeast (blue). Procrustes analyses showed a strong and significant correspondence between genetic and geographic structure (*p* < 10^−5^; 10,000 permutations).
**Figure S3:** Individual phylogeny reconstructed from neutral, unlinked SNPs using IQ‐TREE 2. Colors denote regional lineages inferred from population structure analyses: southwest (red), northwest (yellow), southeast (green), and northeast (blue). Euthalia thibetana (sample N1_4351; shown in black) was included as the outgroup following Toussaint et al. (2020). Branch thickness corresponds to bootstrap support, with the thickest branches indicating support values greater than 70.
**Figure S4:** Effective migration surfaces of Euthalia formosana estimated using FEEMS. (A) Effective migration surfaces generated under four combinations of regularization parameters. Higher values (blue) represent regions of elevated effective migration, whereas lower values (brown) indicate reduced effective migration and potential barriers to gene flow. Parameter settings: a = λ = 100, λ_q = 10; b = λ = 0.17, λ_q = 10; c = λ = 0.17, λ_q = 0.1; d = λ = 0, λ_q = 0.1. Point color indicate regional lineages: southwest (red), northwest (yellow), southeast (green), and northeast (blue). Under the K = 3 configuration, the southeast and northeast lineages merge into a single eastern lineage. (B) Cross‐validation error across parameter combinations, with Model b identified as the best‐supported configuration. (C) Predicted versus observed genetic distances under the best‐supported model (Model b), illustrating the strong fit between FEEMS‐inferred connectivity and empirical genomic data.
**Figure S5:** Genetic diversity of all sampled Euthalia formosana individuals across Taiwan. Multilocus heterozygosity is shown for each individual. Colors denote regional lineages: southwest (red), northwest (yellow), southeast (green), and northeast (blue).
**Figure S6:** Long‐term demographic history of the four lineages of Euthalia formosana across Taiwan. Stairway Plot reconstructions of effective population size (Ne) through time for the southwest (red), northwest (yellow), southeast (green), and northeast (blue) lineages. Dashed regions (a) and (b) indicate two major periods of population decline shared by all four lineages: (a) an early decline occurring approximately 2–4.5 kya, and (b) a more recent decline occurring approximately 0.2–0.45 kya.
**Figure S7:** Summary of key environmental predictors and environmental clustering. (A) Relative importance of the four bioclimatic variables retained after the mtry‐based filtering procedure. Temperature‐related variables are shown in red, and precipitation‐related variables are shown in blue. (B) Dendrogram illustrating relationships among the five environmental clusters identified by turnover‐curve clustering across the landscape of Taiwan.
**Figure S8:** Projections of genetic offset under SSP126 for the four lineages of Euthalia formosana. Point colors indicate regional lineages: southwest (red), northwest (yellow), southeast (green), and northeast (blue). Genetic offset values represent the magnitude of genomic change required for populations to remain environmentally matched under future conditions, with darker colors indicating greater deviation from current conditions. Populations for which all individuals fall outside the projected future environmental domain used to calibrate the model are marked as non‐analog (question mark). Panels show predicted genetic offset for (A) 2021–2040, (B) 2041–2060, (C) 2061–2080, and (D) 2081–2100.
**Figure S9:** Projections of genetic offset under SSP370 for the four lineages of Euthalia formosana. Point colors indicate regional lineages: southwest (red), northwest (yellow), southeast (green), and northeast (blue). Genetic offset values represent the magnitude of genomic change required for populations to remain environmentally matched under future conditions, with darker colors indicating greater deviation from current conditions. Populations for which all individuals fall outside the projected future environmental domain used to calibrate the model are marked as non‐analog (question mark). Panels show predicted genetic offset for (A) 2021–2040, (B) 2041–2060, (C) 2061–2080, and (D) 2081–2100.
**Figure S10:** Projections of genetic offset under SSP585 for the four lineages of Euthalia formosana. Point colors indicate regional lineages: southwest (red), northwest (yellow), southeast (green), and northeast (blue). Genetic offset values represent the magnitude of genomic change required for populations to remain environmentally matched under future conditions, with darker colors indicating greater deviation from current conditions. Populations for which all individuals fall outside the projected future environmental domain used to calibrate the model are marked as non‐analog (question mark). Panels show predicted genetic offset for (A) 2021–2040, (B) 2041–2060, (C) 2061–2080, and (D) 2081–2100.
**Table S1:** Number of R80 loci identified under different M/n settings for the full dataset and the Euthalia formosana–only dataset.

## Data Availability

The raw sequence reads have been deposited in the NCBI Sequence Read Archive (SRA) under BioProject PRJNA1403191. Accession numbers for each individual are provided in the Data S1.

## References

[eva70309-bib-0001] Aguilar‐Gómez, D. , L. Yuan , Y. Zhang , et al. 2025. “Genetic Rescue of Florida Panthers Reduced Homozygosity but Did Not Swamp Ancestral Genotypes.” Proceedings of the National Academy of Sciences of the United States of America 122, no. 31: e2410945122. 10.1073/pnas.2410945122. 40720660 PMC12337334

[eva70309-bib-0002] Alexander, D. H. , J. Novembre , and K. Lange . 2009. “Fast Model‐Based Estimation of Ancestry in Unrelated Individuals.” Genome Research 19: 1655–1664. 10.1101/gr.094052.109. 19648217 PMC2752134

[eva70309-bib-0003] Ali, J. R. , and U. Fritz . 2025. “Taiwan's Land Vertebrate Suite: An Assemblage Forged by Tectonism and Sea‐Level Shifts.” Palaeogeography, Palaeoclimatology, Palaeoecology 684: 113502. 10.1016/j.palaeo.2025.113502.

[eva70309-bib-0004] Allaire, J. J. , C. Gandrud , K. Russell , and C. J. Yetman . 2017. “networkD3: D3 JavaScript Network Graphs From R. R Package Version 0.4.” https://CRAN.R‐project.org/package=networkD3.

[eva70309-bib-0005] Beale, C. M. , and J. J. Lennon . 2012. “Incorporating Uncertainty in Predictive Species Distribution Modelling.” Philosophical Transactions of the Royal Society, B: Biological Sciences 367: 247–258. 10.1098/rstb.2011.0178. PMC3223803 22144387

[eva70309-bib-0006] Beugin, M. P. , T. Gayet , D. Pontier , S. Devillard , and T. Jombart . 2018. “A Fast Likelihood Solution to the Genetic Clustering Problem.” Methods in Ecology and Evolution 9, no. 4: 1006–1016. 10.1111/2041-210X.12968. 29938015 PMC5993310

[eva70309-bib-0007] Catchen, J. , P. A. Hohenlohe , S. Bassham , A. Amores , and W. A. Cresko . 2013. “Stacks: An Analysis Tool Set for Population Genomics.” Molecular Ecology 22, no. 11: 3124–3140. 10.1111/mec.12354. 23701397 PMC3936987

[eva70309-bib-0008] Chen, Y.‐Y. , W. Huang , W.‐H. Wang , et al. 2019. “Reconstructing Taiwan's Land Cover Changes Between 1904 and 2015 From Historical Maps and Satellite Images.” Scientific Reports 9: 3643. 10.1038/s41598-019-40063-1. 30842476 PMC6403323

[eva70309-bib-0009] Chen, Z. , L. Grossfurthner , J. L. Loxterman , et al. 2022. “Applying Genomics in Assisted Migration Under Climate Change: Framework, Empirical Applications, and Case Studies.” Evolutionary Applications 15, no. 1: 3–21. 10.1111/eva.13335. 35126645 PMC8792483

[eva70309-bib-0010] Chiang, Y.‐C. , B.‐H. Huang , and P.‐C. Liao . 2012. “Diversification, Biogeographic Pattern, and Demographic History of Taiwanese *Scutellaria* Species Inferred From Nuclear and Chloroplast DNA.” PLoS One 7, no. 11: e50844. 10.1371/journal.pone.0050844. 23226402 PMC3511331

[eva70309-bib-0011] Chifman, J. , and L. Kubatko . 2014. “Quartet Inference From SNP Data Under the Coalescent Model.” Bioinformatics 30, no. 23: 3317–3324. 10.1093/bioinformatics/btu530. 25104814 PMC4296144

[eva70309-bib-0012] Coltman, D. W. , J. G. Pilkington , J. A. Smith , and J. M. Pemberton . 1999. “Parasite‐Mediated Selection Against Inbred Soay Sheep in a Free‐Living Island Population.” Evolution 53: 1259–1267. 10.1111/j.1558-5646.1999.tb04538.x. 28565537

[eva70309-bib-0013] Côte, J. , J.‐M. Roussel , S. Le Cam , and G. Evanno . 2014. “Outbreeding Depression in Atlantic Salmon Revealed by Hypoxic Stress During Embryonic Development.” Evolutionary Biology 41: 561–571. 10.1007/s11692-014-9289-0.

[eva70309-bib-0014] David, P. , B. Pujol , F. Viard , V. Castella , and J. Goudet . 2007. “Reliable Selfing Rate Estimates From Imperfect Population Genetic Data.” Molecular Ecology 16, no. 12: 2474–2487. 10.1111/j.1365-294X.2007.03330.x. 17561907

[eva70309-bib-0015] De Vivo, M. , M. H. Chou , S. P. Wu , et al. 2023. “Genomic Tools for Comparative Conservation Genetics Among Three Recently Diverged Stag Beetles (*Lucanus*, Lucanidae).” Insect Conservation and Diversity 16, no. 6: 853–869. 10.1111/icad.12678.

[eva70309-bib-0016] Dixon, P. 2003. “VEGAN, a Package of R Functions for Community Ecology.” Journal of Vegetation Science 14, no. 6: 927–930. 10.1111/j.1654-1103.2003.tb02228.x.

[eva70309-bib-0017] Dray, S. , and A. B. Dufour . 2007. “The ade4 Package: Implementing the Duality Diagram for Ecologists.” Journal of Statistical Software 22, no. 4: 1–20. 10.18637/jss.v022.i04.

[eva70309-bib-0018] Fick, S. E. , and R. J. Hijmans . 2017. “WorldClim 2: New 1‐Km Spatial Resolution Climate Surfaces for Global Land Areas.” International Journal of Climatology 37, no. 12: 4302–4315. 10.1002/joc.5086.

[eva70309-bib-0019] Fitzpatrick, M. C. , and W. W. Hargrove . 2009. “The Projection of Species Distribution Models and the Problem of Non‐Analog Climate.” Biodiversity and Conservation 18: 2255–2261. 10.1007/s10531-009-9584-8.

[eva70309-bib-0020] Fitzpatrick, M. C. , and S. R. Keller . 2015. “Ecological Genomics Meets Community‐Level Modelling of Biodiversity: Mapping the Genomic Landscape of Current and Future Environmental Adaptation.” Ecology Letters 18, no. 1: 1–16. 10.1111/ele.12376. 25270536

[eva70309-bib-0021] Frankham, R. , J. D. Ballou , M. D. B. Eldridge , et al. 2011. “Predicting the Probability of Outbreeding Depression.” Conservation Biology 25, no. 3: 465–475. 10.1111/j.1523-1739.2011.01662.x. 21486369

[eva70309-bib-0022] Frichot, E. , and O. François . 2015. “LEA: An R Package for Landscape and Ecological Association Studies.” Methods in Ecology and Evolution 6, no. 8: 925–929. 10.1111/2041-210X.12382.

[eva70309-bib-0023] Frichot, E. , F. Mathieu , T. Trouillon , G. Bouchard , and O. François . 2014. “Fast and Efficient Estimation of Individual Ancestry Coefficients.” Genetics 196, no. 4: 973–983. 10.1534/genetics.113.160572. 24496008 PMC3982712

[eva70309-bib-0024] Gu, Z. , L. Gu , R. Eils , M. Schlesner , and B. Brors . 2014. “ *Circlize* Implements and Enhances Circular Visualization in R.” Bioinformatics 30, no. 19: 2811–2812. 10.1093/bioinformatics/btu393. 24930139

[eva70309-bib-0025] Guindon, S. , J.‐F. Dufayard , V. Lefort , M. Anisimova , W. Hordijk , and O. Gascuel . 2010. “New Algorithms and Methods to Estimate Maximum‐Likelihood Phylogenies: Assessing the Performance of PhyML 3.0.” Systematic Biology 59, no. 3: 307–321. 10.1093/sysbio/syq010. 20525638

[eva70309-bib-0026] Gutenkunst, R. N. , R. D. Hernandez , S. H. Williamson , and C. D. Bustamante . 2009. “Inferring the Joint Demographic History of Multiple Populations From Multidimensional SNP Frequency Data.” PLoS Genetics 5, no. 10: e1000695. 10.1371/journal.pgen.1000695. 19851460 PMC2760211

[eva70309-bib-0027] He, J. , Z. Gao , Y. Su , S. Lin , and H. Jiang . 2018. “Geographical and Temporal Origins of Terrestrial Vertebrates Endemic to Taiwan.” Journal of Biogeography 45: 2458–2470. 10.1111/jbi.13438.

[eva70309-bib-0028] Hoang, D. T. , O. Chernomor , A. von Haeseler , B. Q. Minh , and L. S. Vinh . 2018. “UFBoot2: Improving the Ultrafast Bootstrap Approximation.” Molecular Biology and Evolution 35, no. 2: 518–522. 10.1093/molbev/msx281. 29077904 PMC5850222

[eva70309-bib-0029] Hoffman, J. I. , F. Simpson , P. David , et al. 2014. “High‐Throughput Sequencing Reveals Inbreeding Depression in a Natural Population.” Proceedings of the National Academy of Sciences 111, no. 10: 3775–3780. 10.1073/pnas.1318945111. PMC3956162 24586051

[eva70309-bib-0030] Hsieh, C. F. , I. L. Lai , G. Z. Song , C. C. Liao , and K. C. Yang . 1996. “Biodiversity and Conservation of the Evergreen Broad‐Leaved Forests in Taiwan.” Tropics 6: 361–370. 10.3759/tropics.6.361.

[eva70309-bib-0031] Hsu, Y. F. 2003. Butterflies of Taiwan. Vol. II. Fonghuanggu Bird and Ecology Park.

[eva70309-bib-0032] Hsu, Y. F. 2006. The Study of Molecular Phylogeny and Genetic Diversity of Euthalini in Taiwan (I) (in Chinese). Council of Agriculture, Executive Yuan.

[eva70309-bib-0033] Hsu, Y. F. , H. Chiba , H. Tsukiyama , J. Y. Liang , and C. W. Huang . 2019. Butterfly Fauna of Taiwan. Vol. III: Hesperidae. Council of Agriculture, Executive Yuan.

[eva70309-bib-0034] Hsu, Y. F. , C. L. Huang , and J. Y. Liang . 2018a. Butterfly Fauna of Taiwan. Vol. I: Papilionidae. Council of Agriculture, Executive Yuan.

[eva70309-bib-0035] Hsu, Y. F. , C. L. Huang , and J. Y. Liang . 2018b. Butterfly Fauna of Taiwan. Vol. II: Pieridae. Council of Agriculture, Executive Yuan.

[eva70309-bib-0036] Hsu, Y. F. , J. Y. Liang , and C. W. Huang . 2020. Butterfly Fauna of Taiwan. Vol. IV: Lycaenidae. Council of Agriculture, Executive Yuan.

[eva70309-bib-0037] Hsu, Y. F. , J. Y. Liang , C. W. Huang , and Z. Y. Shen . 2021. Butterfly Fauna of Taiwan. Vol. V: Nymphalidae. Council of Agriculture, Executive Yuan.

[eva70309-bib-0038] Huang, J.‐P. , and C.‐P. Lin . 2010. “Diversification in Subtropical Mountains: Phylogeography, Pleistocene Demographic Expansion, and Evolution of Polyphenic Mandibles in the Taiwanese Stag Beetle *Lucanus formosanus* .” Molecular Phylogenetics and Evolution 57: 1149–1161. 10.1016/j.ympev.2010.10.012. 20971199

[eva70309-bib-0039] Huang, J.‐P. , and C.‐P. Lin . 2011. “Lineage‐Specific Late Pleistocene Expansion of an Endemic Subtropical Gossamer‐Wing Damselfly, *Euphaea formosa*, in Taiwan.” BMC Evolutionary Biology 11: 94. 10.1186/1471-2148-11-94. 21486452 PMC3094233

[eva70309-bib-0040] Huang, J.‐P. , S.‐P. Wu , W.‐Y. Chen , G. J. Pham , and Y.‐H. Kuan . 2024. “Genomic Data Revealed Inbreeding Despite a Geographically Connected Stable Effective Population Size Since the Holocene in the Protected Formosan Long‐Arm Scarab Beetle, *Cheirotonus formosanus* .” Journal of Heredity 115, no. 3: 292–301. 10.1093/jhered/esae006. 38364316

[eva70309-bib-0041] Johnson, W. E. , D. P. Onorato , M. E. Roelke , et al. 2010. “Genetic Restoration of the Florida Panther.” Science 329: 1641–1645. 10.1126/science.1192891. 20929847 PMC6993177

[eva70309-bib-0042] Jombart, T. 2008. “Adegenet: A R Package for the Multivariate Analysis of Genetic Markers.” Bioinformatics 24, no. 11: 1403–1405. 10.1093/bioinformatics/btn129. 18397895

[eva70309-bib-0043] Kalyaanamoorthy, S. , B. Q. Minh , T. K. F. Wong , A. von Haeseler , and L. S. Jermiin . 2017. “ModelFinder: Fast Model Selection for Accurate Phylogenetic Estimates.” Nature Methods 14: 587–589. 10.1038/nmeth.4285. 28481363 PMC5453245

[eva70309-bib-0044] Kassambara, A. , and F. Mundt . 2020. “factoextra: Extract and Visualize the Results of Multivariate Data Analyses. R Package, Version 1.0.7.” https://CRAN.R‐project.org/package=factoextra.

[eva70309-bib-0045] Keightley, P. D. , A. Pinharanda , R. W. Ness , et al. 2015. “Estimation of the Spontaneous Mutation Rate in *Heliconius melpomene* .” Molecular Biology and Evolution 32, no. 1: 239–243. 10.1093/molbev/msu302. 25371432 PMC4271535

[eva70309-bib-0046] Knaus, B. J. , and N. J. Grünwald . 2017. “Vcfr: A Package to Manipulate and Visualize Variant Call Format Data in R.” Molecular Ecology Resources 17, no. 1: 44–53. 10.1111/1755-0998.12549. 27401132

[eva70309-bib-0047] Knowles, L. L. , R. Massatti , Q. He , L. E. Olson , and H. C. Lanier . 2016. “Quantifying the Similarity Between Genes and Geography Across Alaska's Alpine Small Mammals.” Journal of Biogeography 43: 1464–1476. 10.1111/jbi.12728.

[eva70309-bib-0048] Kruskal, W. H. , and W. A. Wallis . 1952. “Use of Ranks in One‐Criterion Variance Analysis.” Journal of the American Statistical Association 47, no. 260: 583–621. 10.1080/01621459.1952.10483441.

[eva70309-bib-0049] Łabiszak, B. , and W. Wachowiak . 2025. “Adaptive Potential and Genomic Vulnerability of Keystone Forest Tree Species to Climate Change: A Case Study in Scots Pine.” Evolutionary Applications 18, no. 12: e70180. 10.1111/eva.70180. 41357564 PMC12680428

[eva70309-bib-0050] Lee, Y.‐C. , Z.‐Y. Shen , W.‐R. Lin , et al. 2025. “Eco‐Genomic Analysis Uncovers Precision‐Conservation Targets for the Western Pacific's Southernmost Salmonid.” bioRxiv., Preprint. 10.1101/2025.09.09.675276.

[eva70309-bib-0051] Li, C.‐F. , M. Chytrý , D. Zelený , et al. 2013. “Classification of Taiwan Forest Vegetation.” Applied Vegetation Science 16: 698–719. 10.1111/avsc.12025.

[eva70309-bib-0052] Lin, H.‐C. , S.‐H. Li , J. Fong , and S. M. Lin . 2008. “Ventral Coloration Differentiation and Mitochondrial Sequences of the Chinese Cobra ( *Naja atra* ) in Taiwan.” Conservation Genetics 9: 1089–1097. 10.1007/s10592-007-9418-8.

[eva70309-bib-0053] Lin, H.‐D. , Y.‐R. Chen , and S.‐M. Lin . 2012. “Strict Consistency Between Genetic and Topographic Landscapes of the Brown Tree Frog ( *Buergeria robusta* ) in Taiwan.” Molecular Phylogenetics and Evolution 62: 251–262. 10.1016/j.ympev.2011.09.022. 22019937

[eva70309-bib-0054] Lin, H.‐H. , and L.‐C. Shih . 2025. Taiwan Moth Occurrence Data Collected From Social Network. Version 1.104. Taiwan Biodiversity Research Institute. 10.15468/3qxzlj.

[eva70309-bib-0055] Lin, T.‐H. , Z.‐Y. Shen , M.‐H. Chou , et al. 2025. “Allopatric Speciation and Interspecific Gene Flow Driven by Niche Conservatism of *Diploderma* Tree Lizards in Taiwan.” Molecular Ecology 34, no. 8: e17718. 10.1111/mec.17718. 40052357

[eva70309-bib-0056] Lin, Y.‐P. , C.‐Y. Lu , and C.‐R. Lee . 2023. “The Past Contribution and Future Fate of Genetic Variants Under Climate Change in an Island Population of *Musa itinerans* .” American Naturalist 202, no. 4: 558–570. 10.1086/726015. 37792919

[eva70309-bib-0057] Liu, T.‐J. 2019. Environmental History of Taiwan. National Taiwan University Press.

[eva70309-bib-0058] Liu, X. , and Y. X. Fu . 2020. “Stairway Plot 2: Demographic History Inference With Folded SNP Frequency Spectra.” Genome Biology 21: 280. 10.1186/s13059-020-02196-9. 33203475 PMC7670622

[eva70309-bib-0059] Marcus, J. , W. Ha , R. F. Barber , and J. Novembre . 2021. “Fast and Flexible Estimation of Effective Migration Surfaces.” eLife 10: e61927. 10.7554/eLife.61927. 34328078 PMC8324296

[eva70309-bib-0060] Mastretta‐Yanes, A. , N. Arrigo , N. Alvarez , T. H. Jorgensen , D. Piñero , and B. C. Emerson . 2015. “Restriction Site‐Associated DNA Sequencing, Genotyping Error Estimation and De Novo Assembly Optimization for Population Genetic Inference.” Molecular Ecology Resources 15, no. 1: 28–41. 10.1111/1755-0998.12291. 24916682

[eva70309-bib-0061] Matz, M. V. , and K. L. Black . 2025. “RDAforest: Identifying Environmental Drivers of Polygenic Adaptation.” Molecular Ecology Resources 25, no. 8: e70002. 10.1111/1755-0998.70002. 40616287 PMC12550478

[eva70309-bib-0062] Minh, B. Q. , M. A. T. Nguyen , and A. von Haeseler . 2013. “Ultrafast Approximation for Phylogenetic Bootstrap.” Molecular Biology and Evolution 30, no. 5: 1188–1195. 10.1093/molbev/mst024. 23418397 PMC3670741

[eva70309-bib-0063] Minh, B. Q. , H. A. Schmidt , O. Chernomor , et al. 2020. “IQ‐TREE 2: New Models and Efficient Methods for Phylogenetic Inference in the Genomic Era.” Molecular Biology and Evolution 37, no. 5: 1530–1534. 10.1093/molbev/msaa015. 32011700 PMC7182206

[eva70309-bib-0064] Moritz, C. , and R. Agudo . 2013. “The Future of Species Under Climate Change: Resilience or Decline?” Science 341, no. 6145: 504–508. 10.1126/science.1237190. 23908228

[eva70309-bib-0065] Mussmann, S. M. , M. R. Douglas , T. K. Chafin , and M. E. Douglas . 2019. “BA3‐SNPs: Contemporary Migration Reconfigured in BayesAss for Next‐Generation Sequence Data.” Methods in Ecology and Evolution 10, no. 10: 1808–1813. 10.1111/2041-210X.13252.

[eva70309-bib-0066] Nagel, L. M. , B. J. Palik , M. A. Battaglia , et al. 2017. “Adaptive Silviculture for Climate Change: A National Experiment in Manager–Scientist Partnerships to Apply an Adaptation Framework.” Journal of Forestry 115: 167–178. 10.5849/jof.16-039.

[eva70309-bib-0067] Ortiz, E. M. 2019. vcf2phylip v2.0: Convert VCF to PHYLIP and Other Alignment Formats. Zenodo. 10.5281/zenodo.2540861.

[eva70309-bib-0068] Otto‐Bliesner, B. L. , S. J. Marshall , J. T. Overpeck , G. H. Miller , A. Hu , and CAPE Last Interglacial Project members . 2006. “Simulating Arctic Climate Warmth and Icefield Retreat in the Last Interglaciation.” Science 311, no. 5768: 1751–1753. 10.1126/science.1120808. 16556838

[eva70309-bib-0069] Paris, J. R. , J. R. Stevens , and J. M. Catchen . 2017. “Lost in Parameter Space: A Road Map for Stacks.” Methods in Ecology and Evolution 8, no. 10: 1360–1373. 10.1111/2041-210X.12775.

[eva70309-bib-0070] Park, A. , and C. Talbot . 2018. “Information Underload: Ecological Complexity, Incomplete Knowledge, and Data Deficits Create Challenges for the Assisted Migration of Forest Trees.” Bioscience 68: 251–263. 10.1093/biosci/biy001.

[eva70309-bib-0071] Parmesan, C. 2006. “Ecological and Evolutionary Responses to Recent Climate Change.” Annual Review of Ecology, Evolution, and Systematics 37: 637–669. 10.1146/annurev.ecolsys.37.091305.110100.

[eva70309-bib-0072] Parmesan, C. , and G. Yohe . 2003. “A Globally Coherent Fingerprint of Climate Change Impacts Across Natural Systems.” Nature 421: 37–42. 10.1038/nature01286. 12511946

[eva70309-bib-0073] Pedlar, J. H. , D. W. McKenney , I. Aubin , et al. 2012. “Placing Forestry in the Assisted Migration Debate.” Bioscience 62: 835–842. 10.1525/bio.2012.62.9.10.

[eva70309-bib-0074] Peterson, B. K. , J. N. Weber , E. H. Kay , H. S. Fisher , and H. E. Hoekstra . 2012. “Double Digest RADseq: An Inexpensive Method for De Novo SNP Discovery and Genotyping in Model and Non‐Model Species.” PLoS One 7: e37135. 10.1371/journal.pone.0037135. 22675423 PMC3365034

[eva70309-bib-0075] Pimm, S. L. , L. Dollar , and O. L. Bass Jr. 2006. “The Genetic Rescue of the Florida Panther.” Animal Conservation 9: 115–122. 10.1111/j.1469-1795.2005.00010.x.

[eva70309-bib-0076] Prates, I. , A. T. Xue , J. L. Brown , et al. 2016. “Inferring Responses to Climate Dynamics From Historical Demography in Neotropical Forest Lizards.” Proceedings of the National Academy of Sciences of the United States of America 113, no. 29: 7978–7985. 10.1073/pnas.1601063113. 27432951 PMC4961184

[eva70309-bib-0077] Purcell, S. , B. Neale , K. Todd‐Brown , et al. 2007. “PLINK: A Toolset for Whole‐Genome Association and Population‐Based Linkage Analyses.” American Journal of Human Genetics 81, no. 3: 559–575. 10.1086/519795. 17701901 PMC1950838

[eva70309-bib-0078] Rambaut, A. , A. J. Drummond , D. Xie , G. Baele , and M. A. Suchard . 2018. “Posterior Summarization in Bayesian Phylogenetics Using Tracer 1.7.” Systematic Biology 67, no. 5: 901–904. 10.1093/sysbio/syy032. 29718447 PMC6101584

[eva70309-bib-0079] Rivera‐Colón, A. G. , and J. Catchen . 2022. “Population Genomics Analysis With RAD, Reprised: Stacks.” In Marine Genomics: Methods and Protocols, edited by C. Verde and D. Giordano . Humana Press. 10.1007/978-1-0716-2313-8_7 35727543

[eva70309-bib-0080] Román‐Palacios, C. , and J. J. Wiens . 2020. “Recent Responses to Climate Change Reveal the Drivers of Species Extinction and Survival.” Proceedings of the National Academy of Sciences of the United States of America 117, no. 8: 4211–4217. 10.1073/pnas.1913007117. 32041877 PMC7049143

[eva70309-bib-0081] Shen, X.‐X. , C. Hittinger , and A. Rokas . 2017. “Contentious Relationships in Phylogenomic Studies Can Be Driven by a Handful of Genes.” Nature Ecology & Evolution 1: 0126. 10.1038/s41559-017-0126. PMC5560076 28812701

[eva70309-bib-0082] Shen, Z.‐Y. , M.‐H. Le , M.‐H. Chou , and J.‐P. Huang . 2025. “A Reference Genome Enhances the Power to Detect Signatures of Recent Anthropogenic Impact in Genomic Data: A Lesson Learned From a Stag Beetle System.” BMC Biology 23: 205. 10.1186/s12915-025-02307-7. 40629430 PMC12239466

[eva70309-bib-0083] Shirôzu, T. 1960. Butterflies of Formosa in Colour. Hoikusha.

[eva70309-bib-0084] Stoffel, M. A. , M. Esser , M. Kardos , et al. 2016. “inbreedR: An R Package for the Analysis of Inbreeding Based on Genetic Markers.” Methods in Ecology and Evolution 7, no. 11: 1331–1339. 10.1111/2041-210X.12588.

[eva70309-bib-0085] Swofford, D. L. 2002. PAUP*: Phylogenetic Analysis Using Parsimony (and Other Methods). Version 4. Sinauer Associates.

[eva70309-bib-0086] TaiBIF . 2025. Taiwan Biodiversity Information Facility. TaiBIF Database. https://taibif.tw.

[eva70309-bib-0087] Toussaint, E. F. A. , C. J. Müller , J. Morinière , R. Tänzler , and M. Balke . 2020. “A Glide Over the Indo‐Australian Geological Maze: Repeated Transgressions of Lydekker's and Wallace's Lines in Archdukes, Barons and Dukes (Nymphalidae: Limenitidinae: Adoliadini).” Biological Journal of the Linnean Society 129, no. 4: 810–821. 10.1093/biolinnean/blaa008.

[eva70309-bib-0088] Waller, D. M. 2015. “Genetic Rescue: A Safe or Risky Bet?” Molecular Ecology 24, no. 11: 2595–2597. 10.1111/mec.13220. 26013990

[eva70309-bib-0089] Walther, G.‐R. , E. Post , P. Convey , et al. 2002. “Ecological Responses to Recent Climate Change.” Nature 416: 389–395. 10.1038/416389a. 11919621

[eva70309-bib-0090] Wang, C. , S. Zöllnerm , and N. A. Rosenberg . 2012. “A Quantitative Comparison of the Similarity Between Gene and Geography in Worldwide Human Populations.” PLoS Genetics 8, no. 8: e1002886. 10.1371/journal.pgen.1002886. 22927824 PMC3426559

[eva70309-bib-0091] Wang, L.‐J. , Y.‐W. Chou , and J.‐P. Huang . 2022. “Testing the Effect of Sampling Effort on Inferring Phylogeographic History in *Psolodesmus mandarinus* (Calopterygidae, Odonata).” Diversity 14, no. 10: 809. 10.3390/d14100809.

[eva70309-bib-0092] Wang, T.‐Y. , T.‐Y. Liao , and C.‐S. Tzeng . 2007. “Phylogeography of the Taiwanese Endemic Hillstream Loaches, *Hemimyzon formosanus* and *H. taitungensis* (Cypriniformes: Balitoridae).” Zoological Studies 46, no. 5: 547–560.

[eva70309-bib-0093] Wei, J.‐H. , C.‐H. Lin , Y.‐F. Hsu , et al. 2025. The Red List of Butterflies in Taiwan. Forestry and Nature Conservation Agency, Council of Agriculture.

[eva70309-bib-0094] White, S. L. , J. M. Rash , and D. C. Kazyak . 2023. “Is Now the Time? Review of Genetic Rescue as a Conservation Tool for Brook Trout Populations.” Ecology and Evolution 13, no. 5: e10142. 10.1002/ece3.10142. 37250443 PMC10213484

[eva70309-bib-0095] Williams, M. I. , and R. K. Dumroese . 2013. “Preparing for Climate Change: Forestry and Assisted Migration.” Journal of Forestry 111: 287–297. 10.5849/jof.13-016.

[eva70309-bib-0096] Wu, T. , Y. Lu , Y. Fang , et al. 2019. “The Beijing Climate Center Climate System Model (BCC‐CSM): The Main Progress From CMIP5 to CMIP6.” Geoscientific Model Development 12: 1573–1600. 10.5194/gmd-12-1573-2019.

[eva70309-bib-0097] Yago, M. , R. Katsuyama , H. Ito , T. Tanio , S. Hoshizaki , and Y. Ishikawa . 2018. “Catalogue of the Insect Collection of Prof. Chûjirô Sasaki and Associated Researchers, the University Museum, the University of Tokyo. Part I (Lepidoptera: Rhopalocera). University Museum Database, the University of Tokyo.” https://umdb.um.u‐tokyo.ac.jp/DEntomology/Sasaki1Rhopalocera/en/index.php.

[eva70309-bib-0098] Yago, M. , R. Katsuyama , Y. Yoshida , and S. Suda . 2023. Catalogue of the Yasunobu Naritomi Butterfly Collection, the University Museum, the University of Tokyo University Museum Database. University of Tokyo. https://umdb.um.u‐tokyo.ac.jp/DEntomology/NaritomiButterfly/en/index.php.

[eva70309-bib-0099] Yan, F. , J. Lü , B. Zhang , et al. 2018. “The Chinese Giant Salamander Exemplifies the Hidden Extinction of Cryptic Species.” Current Biology 28: R590–R592. 10.1016/j.cub.2018.04.004. 29787716

[eva70309-bib-0100] Ye, J. W. , Z. Z. Yang , and B. Tian . 2023. “Tempo‐Spatial Evolution of Seed Plant Endemism in Taiwan Island.” Journal of Biogeography 50: 1981–1991. 10.1111/jbi.14705.

[eva70309-bib-0101] Yeh, L.‐W. , B.‐C. Wang , C.‐C. Lu , C.‐L. Huang , Y.‐F. Hsu , and L.‐W. Wu . 2025. “Integrating Wing Morphometrics and Mitogenomic Sequences Supports Species‐Level Distinction Between *Papilio maraho and Papilio elwesi* (Lepidoptera: Papilionidae).” Zoological Studies 64: 63. 10.6620/ZS.2025.64-63. PMC12835687 41607584

[eva70309-bib-0102] Yoshida, Y. , K. Harada , H. Ito , Y. Ito , and M. Yago . 2015. “‘Catalogue of the Masayo Kato Insect Collection, the University Museum, the University of Tokyo. Part II. Lepidoptera, Rhopalocera’. University Museum Database, the University of Tokyo.” https://umdb.um.u‐tokyo.ac.jp/DEntomology/KatoInsect2/en/index.php.

[eva70309-bib-0103] Zachos, J. C. , M. Pagani , L. Sloan , E. Thomas , and K. Billups . 2001. “Trends, Rhythms, and Aberrations in Global Climate 65 ma to Present.” Science 292, no. 5517: 686–693. 10.1126/science.1059412. 11326091

