# Supplementary material for: Within‐Island Diversification Generates Lineage‐Specific Climate Vulnerability: Insights From the Taiwan‐Endemic Formosan Duke
Source: Evol Appl. 2026 Aug 2;19(8):e70309. doi: 10.1111/eva.70309 (PMC13430063; doi:10.1111/eva.70309)
Supplement: Supplementary file 1 — Data S1: Sampling information and genetic cluster assignments inferred by snapclust. Assignments are shown for K = 3 and K = 4. Figure S1: Selection of the optimal number of lineages (K = 1–8) across population structure analyses. (A) Cross‐validation error for ADMIXTURE clustering solutions. (B) Cross‐entropy values for clustering solutions inferred using sNMF. (C) AIC values for clustering solutions inferred using snapclust. Together, these metrics support selection of the most appropriate number of lineages for downstream analyses. Figure S2: Geographic–genetic correspondence in Euthalia formosana across Taiwan. (A) PCA of individuals grouped under the K = 3 solution. (B) Procrustes comparison of geographic locations (squares) and genetic configurations (circles) under K = 3. Colors: southwest (red), northwest (yellow), east (green). (C) PCA of individuals grouped under the K = 4 solution. (D) Procrustes comparison under K = 4. Colors: southwest (red), northwest (yellow), southeast (green), northeast (blue). Procrustes analyses showed a strong and significant correspondence between genetic and geographic structure (p < 10−5; 10,000 permutations). Figure S3: Individual phylogeny reconstructed from neutral, unlinked SNPs using IQ‐TREE 2. Colors denote regional lineages inferred from population structure analyses: southwest (red), northwest (yellow), southeast (green), and northeast (blue). Euthalia thibetana (sample N1_4351; shown in black) was included as the outgroup following Toussaint et al. (2020). Branch thickness corresponds to bootstrap support, with the thickest branches indicating support values greater than 70. Figure S4: Effective migration surfaces of Euthalia formosana estimated using FEEMS. (A) Effective migration surfaces generated under four combinations of regularization parameters. Higher values (blue) represent regions of elevated effective migration, whereas lower values (brown) indicate reduced effective migration and potential barriers to gen [file EVA-19-e70309-s001.docx]

**
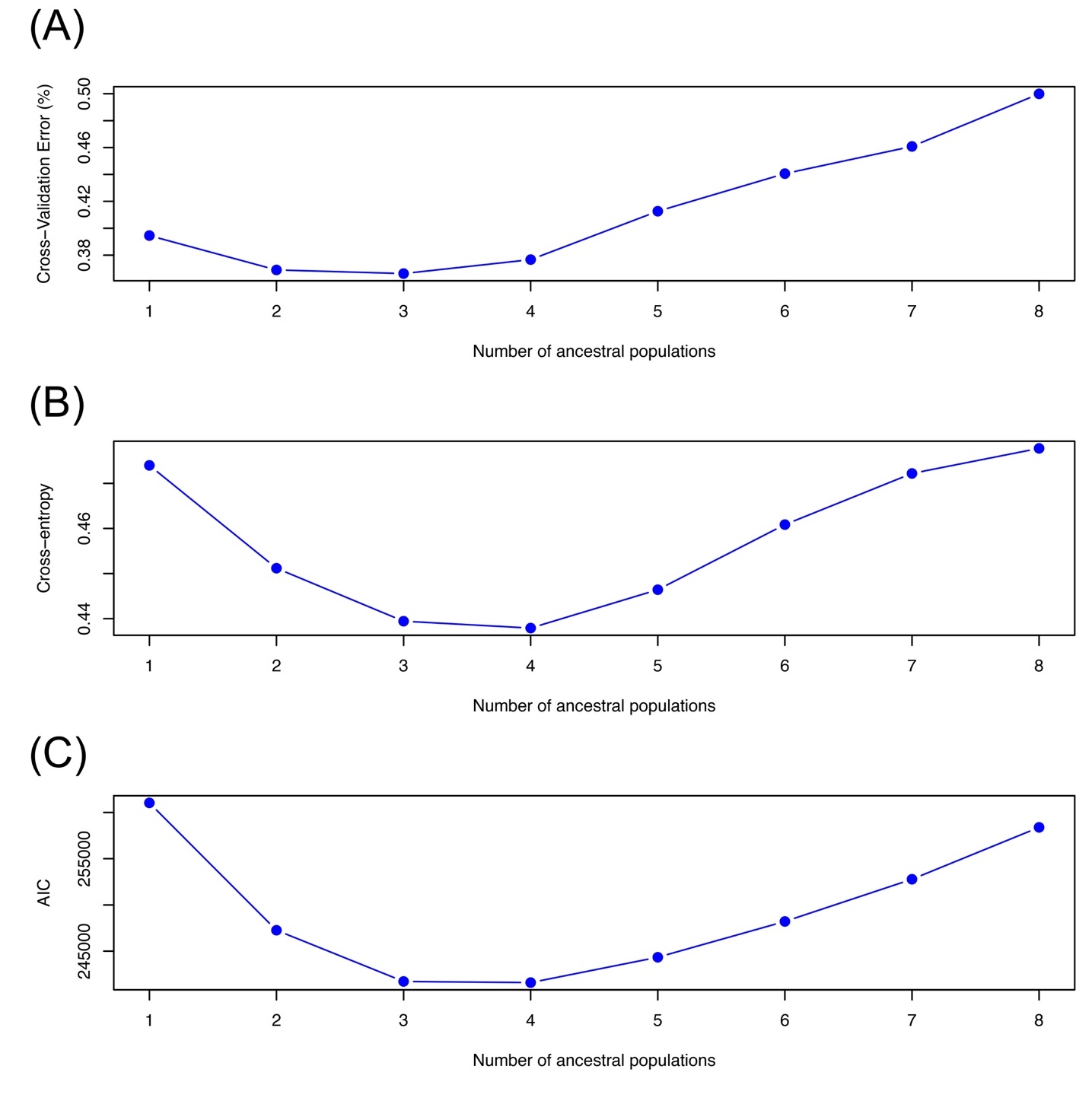
**

**FIGURE S1 |** Selection of the optimal number of lineages (K = 1–8) across population structure analyses. (A) Cross-validation error for ADMIXTURE clustering solutions. (B) Cross-entropy values for clustering solutions inferred using sNMF. (C) AIC values for clustering solutions inferred using snapclust. Together, these metrics support selection of the most appropriate number of lineages for downstream analyses.

**
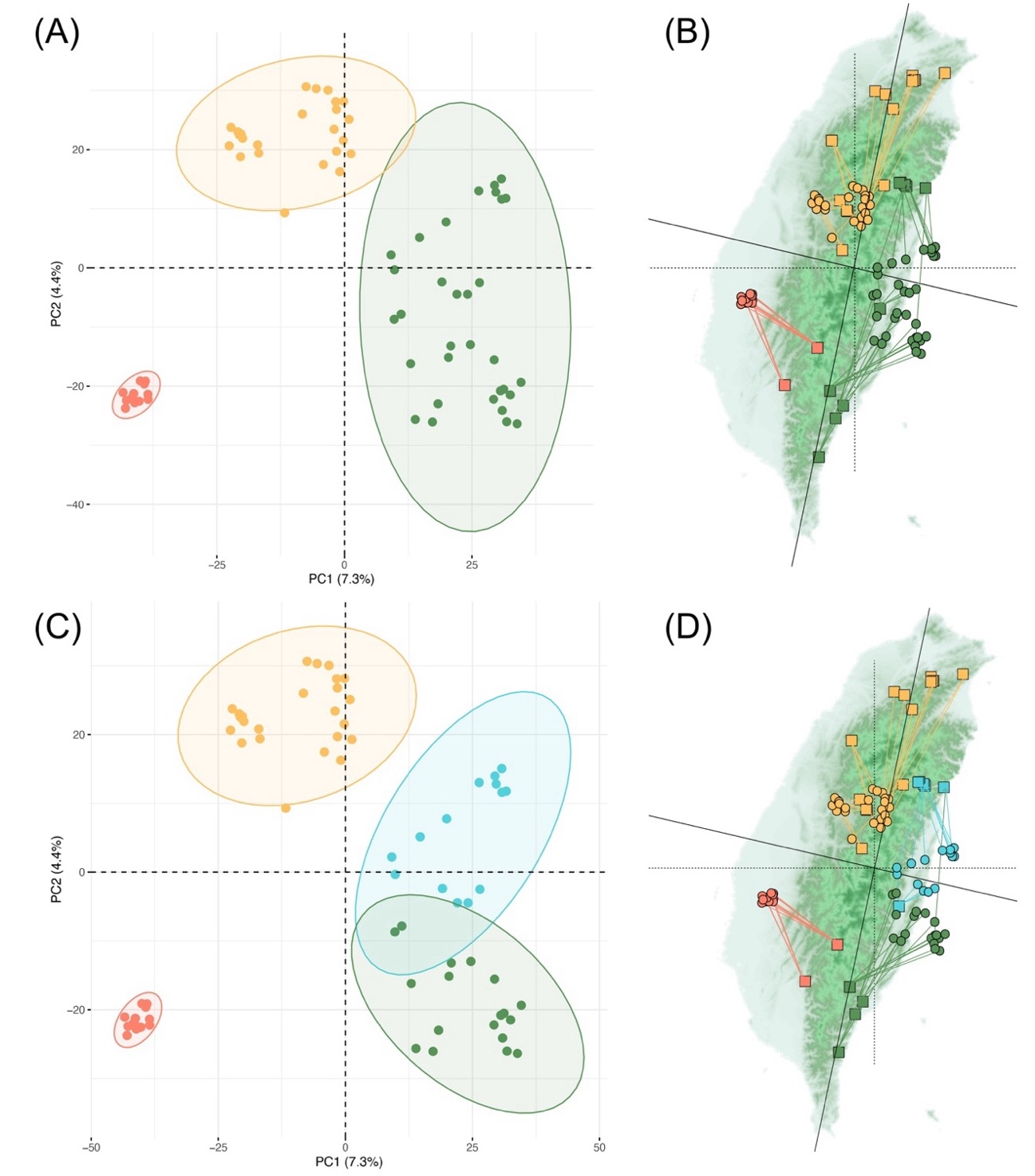
**

**FIGURE S2 |** Geographic–genetic correspondence in *Euthalia formosana* across Taiwan. (A) PCA of individuals grouped under the K = 3 solution. (B) Procrustes comparison of geographic locations (squares) and genetic configurations (circles) under K = 3. Colors: southwest (red), northwest (yellow), east (green). (C) PCA of individuals grouped under the K = 4 solution. (D) Procrustes comparison under K = 4. Colors: southwest (red), northwest (yellow), southeast (green), northeast (blue). Procrustes analyses showed a strong and significant correspondence between genetic and geographic structure (p < 10⁻⁵; 10,000 permutations).


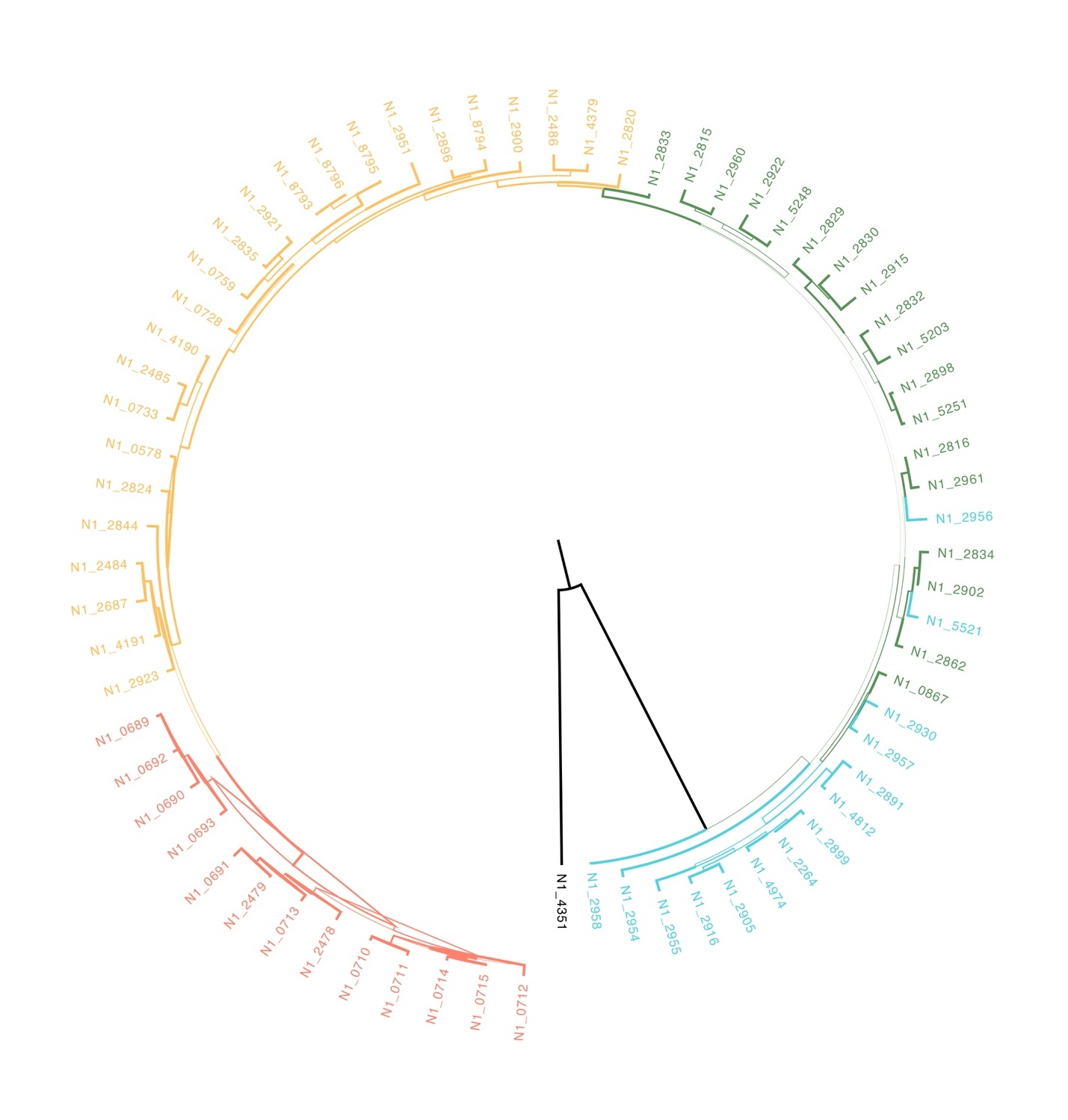


**FIGURE S3 |** Individual phylogeny reconstructed from neutral, unlinked SNPs using IQ-TREE 2. Colors denote regional lineages inferred from population structure analyses: southwest (red), northwest (yellow), southeast (green), and northeast (blue). *Euthalia thibetana* (sample N1_4351; shown in black) was included as the outgroup following Toussaint et al. (2020). Branch thickness corresponds to bootstrap support, with the thickest branches indicating support values greater than 70.

**
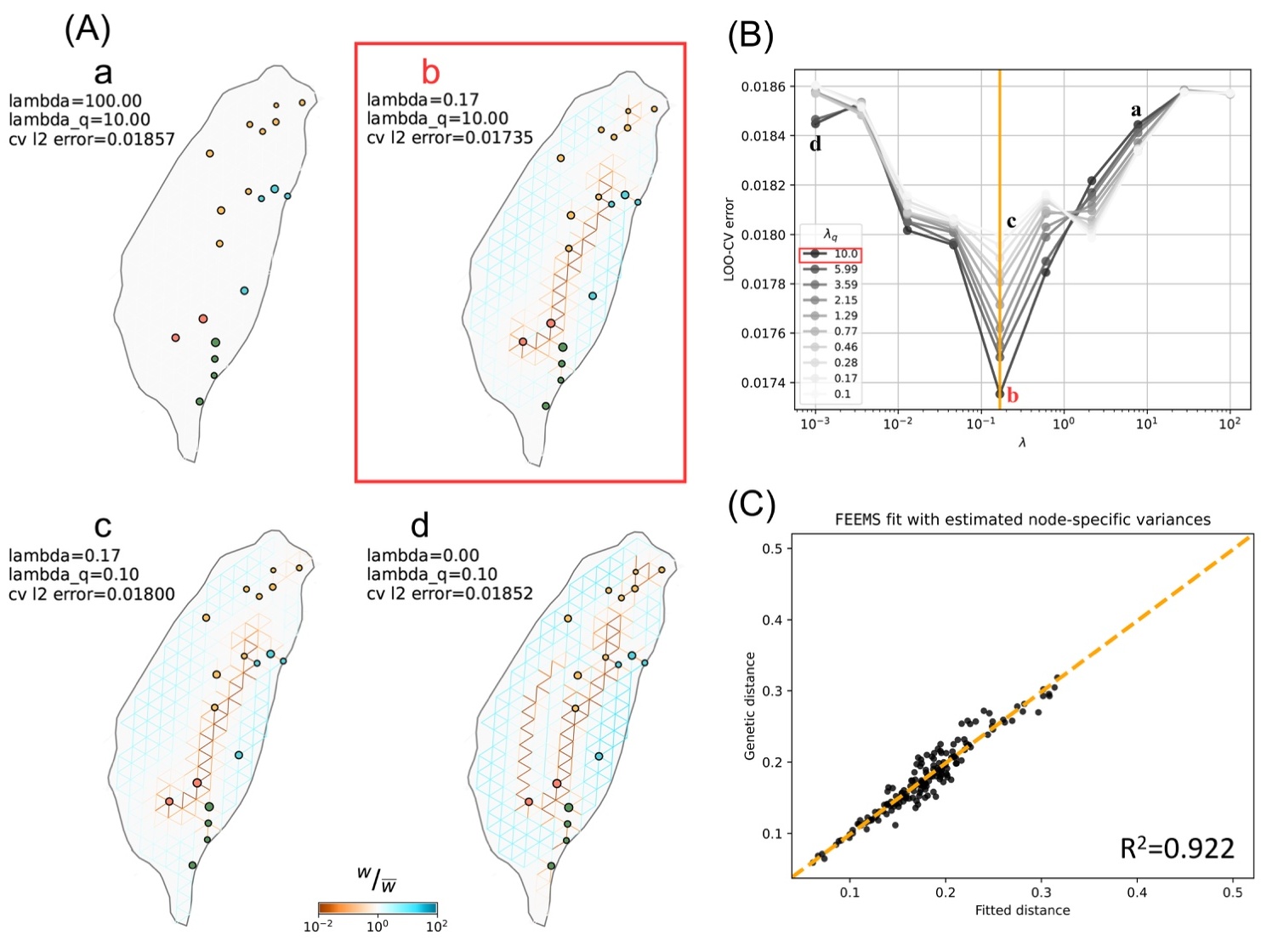
**

**FIGURE S4 |** Effective migration surfaces of *Euthalia formosana* estimated using FEEMS. (A) Effective migration surfaces generated under four combinations of regularization parameters. Higher values (blue) represent regions of elevated effective migration, whereas lower values (brown) indicate reduced effective migration and potential barriers to gene flow. Parameter settings: a = λ = 100, λ_q = 10; b = λ = 0.17, λ_q = 10; c = λ = 0.17, λ_q = 0.1; d = λ = 0, λ_q = 0.1. Point color indicate regional lineages: southwest (red), northwest (yellow), southeast (green), and northeast (blue). Under the K = 3 configuration, the southeast and northeast lineages merge into a single eastern lineage. (B) Cross-validation error across parameter combinations, with Model b identified as the best-supported configuration. (C) Predicted versus observed genetic distances under the best-supported model (Model b), illustrating the strong fit between FEEMS-inferred connectivity and empirical genomic data.


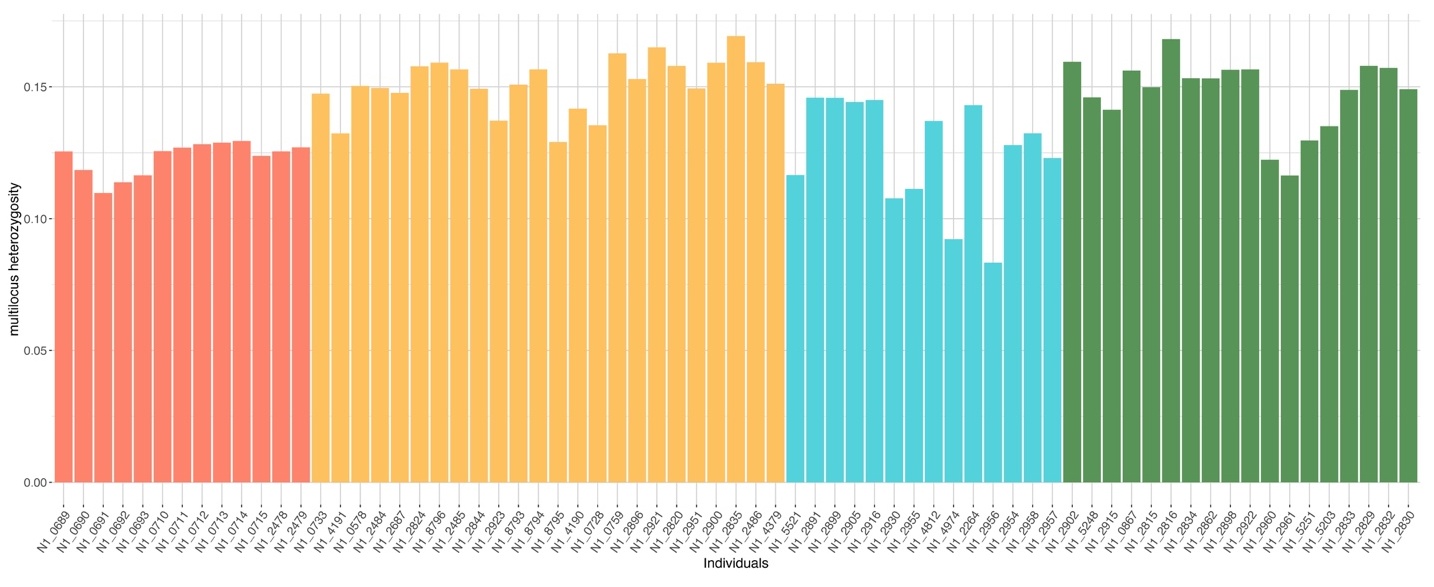


**FIGURE S5 |** Genetic diversity of all sampled *Euthalia formosana* individuals across Taiwan. Multilocus heterozygosity is shown for each individual. Colors denote regional lineages: southwest (red), northwest (yellow), southeast (green), and northeast (blue).

**
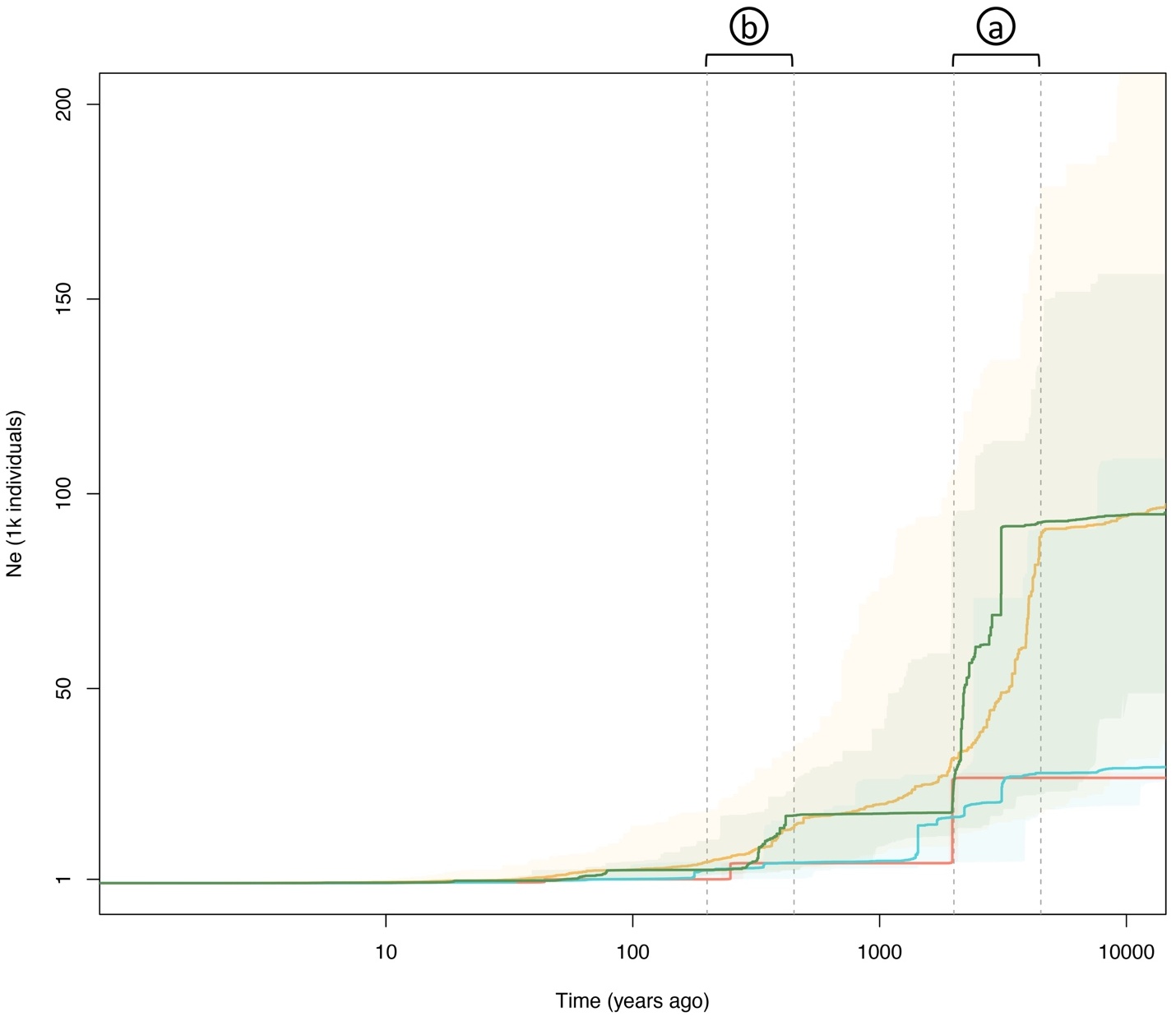
**

**FIGURE S6 |** Long-term demographic history of the four lineages of *Euthalia formosana* across Taiwan. Stairway Plot reconstructions of effective population size (Ne) through time for the southwest (red), northwest (yellow), southeast (green), and northeast (blue) lineages. Dashed regions (a) and (b) indicate two major periods of population decline shared by all four lineages: (a) an early decline occurring approximately 2–4.5 kya, and (b) a more recent decline occurring approximately 0.2–0.45 kya.


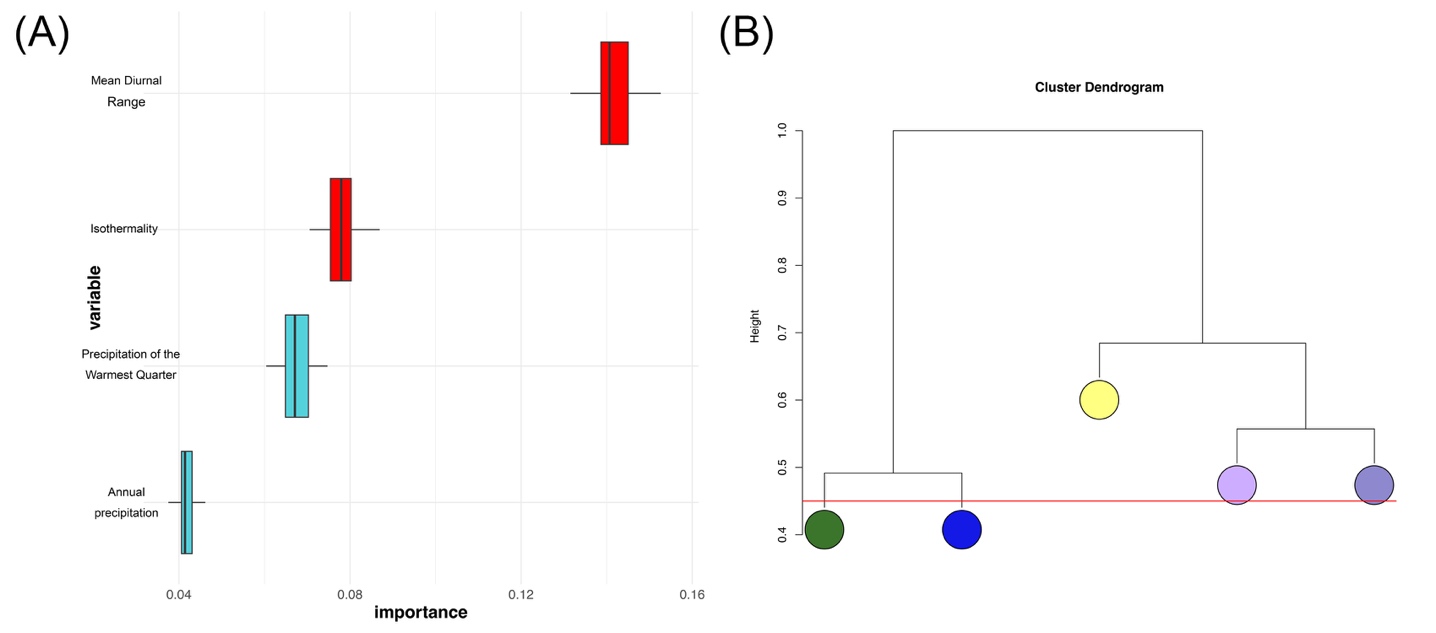


**FIGURE S7 |** Summary of key environmental predictors and environmental clustering. (A) Relative importance of the four bioclimatic variables retained after the *mtry*-based filtering procedure. Temperature-related variables are shown in red, and precipitation-related variables are shown in blue. (B) Dendrogram illustrating relationships among the five environmental clusters identified by turnover-curve clustering across the landscape of Taiwan.


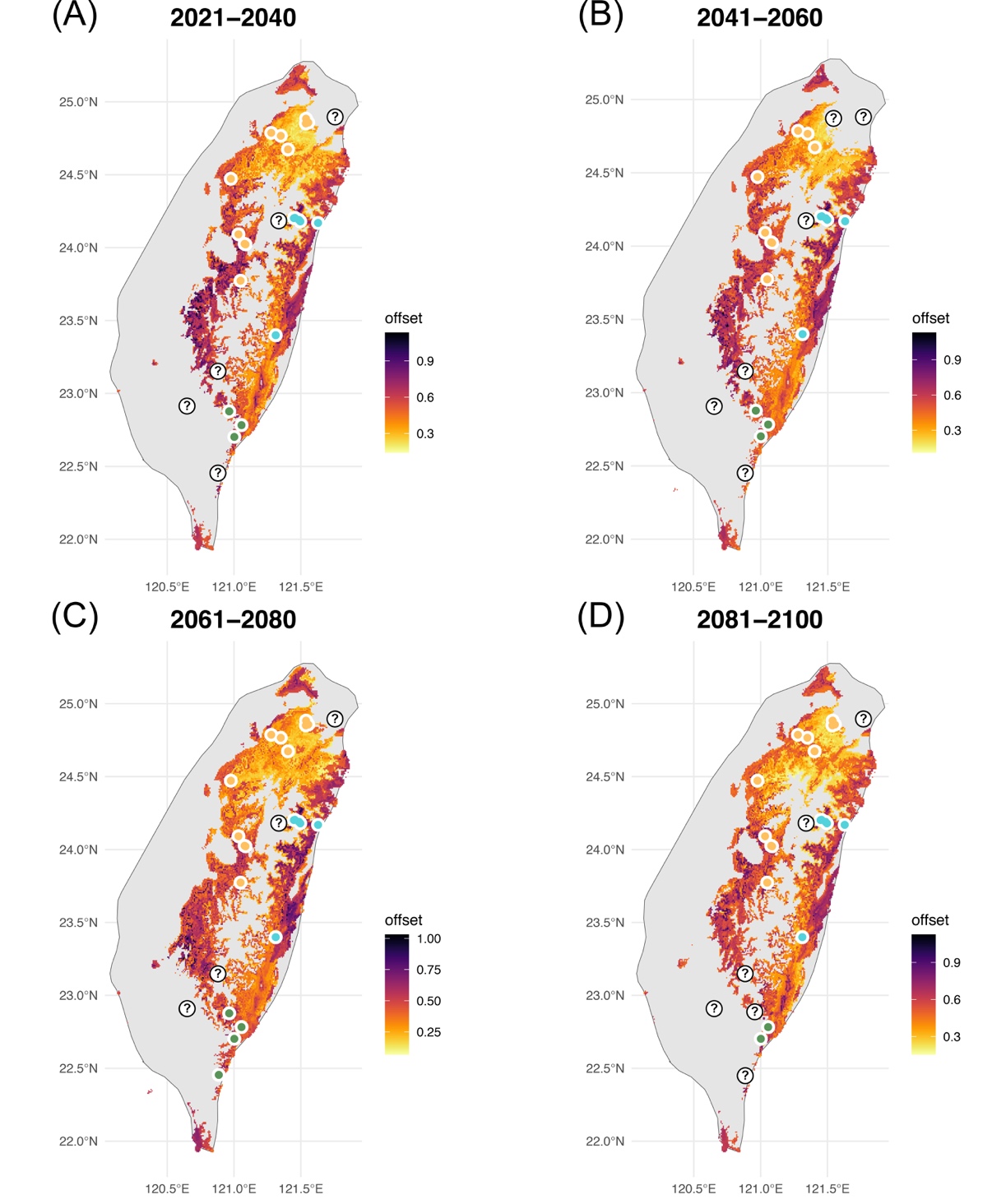


**FIGURE S8 |** Projections of genetic offset under SSP126 for the four lineages of *Euthalia formosana*. Point colors indicate regional lineages: southwest (red), northwest (yellow), southeast (green), and northeast (blue). Genetic offset values represent the magnitude of genomic change required for populations to remain environmentally matched under future conditions, with darker colors indicating greater deviation from current conditions. Populations for which all individuals fall outside the projected future environmental domain used to calibrate the model are marked as non-analog (question mark). Panels show predicted genetic offset for (A) 2021–2040, (B) 2041–2060, (C) 2061–2080, and (D) 2081–2100.


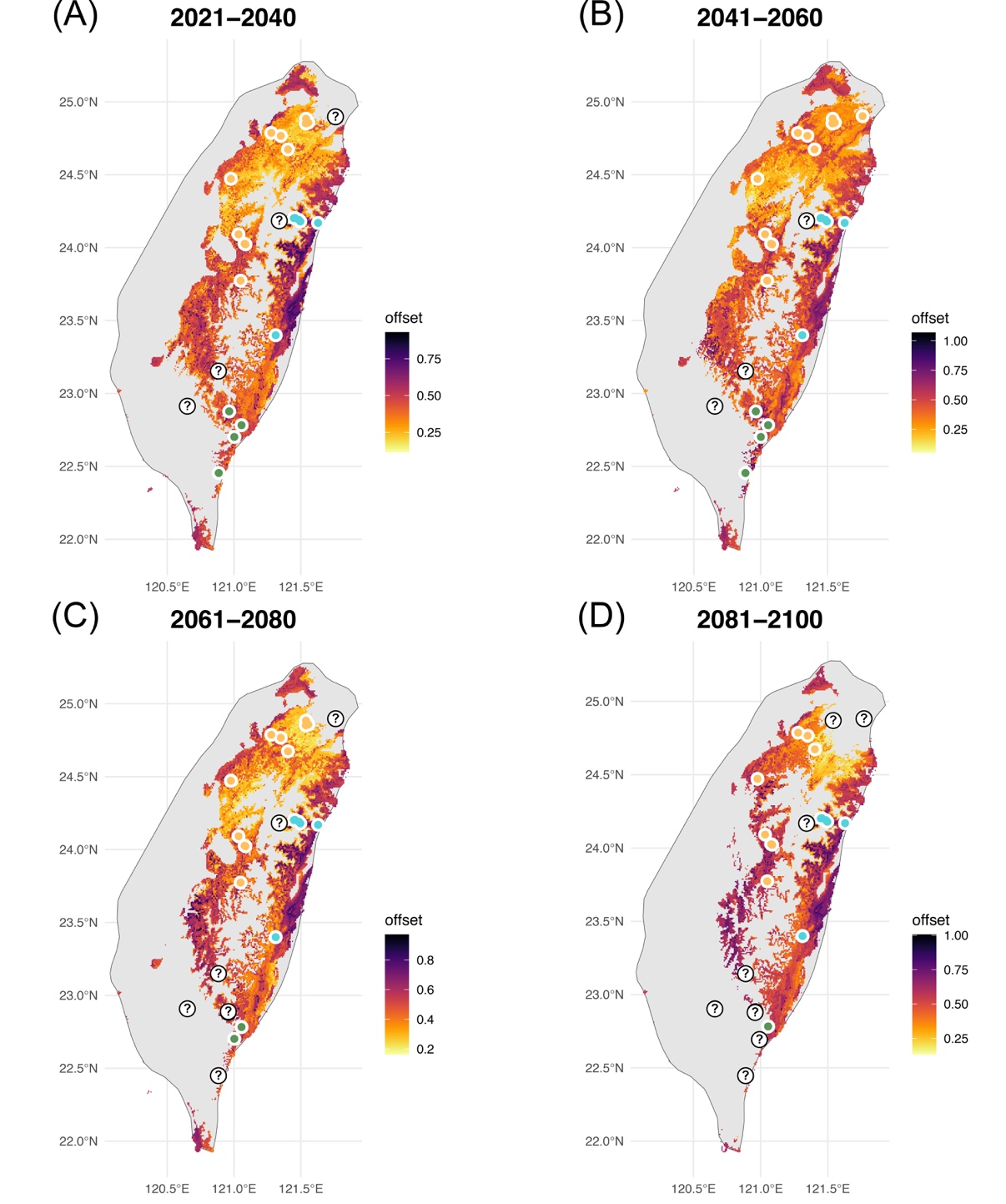


**FIGURE S9 |** Projections of genetic offset under SSP370 for the four lineages of *Euthalia formosana*. Point colors indicate regional lineages: southwest (red), northwest (yellow), southeast (green), and northeast (blue). Genetic offset values represent the magnitude of genomic change required for populations to remain environmentally matched under future conditions, with darker colors indicating greater deviation from current conditions. Populations for which all individuals fall outside the projected future environmental domain used to calibrate the model are marked as non-analog (question mark). Panels show predicted genetic offset for (A) 2021–2040, (B) 2041–2060, (C) 2061–2080, and (D) 2081–2100.


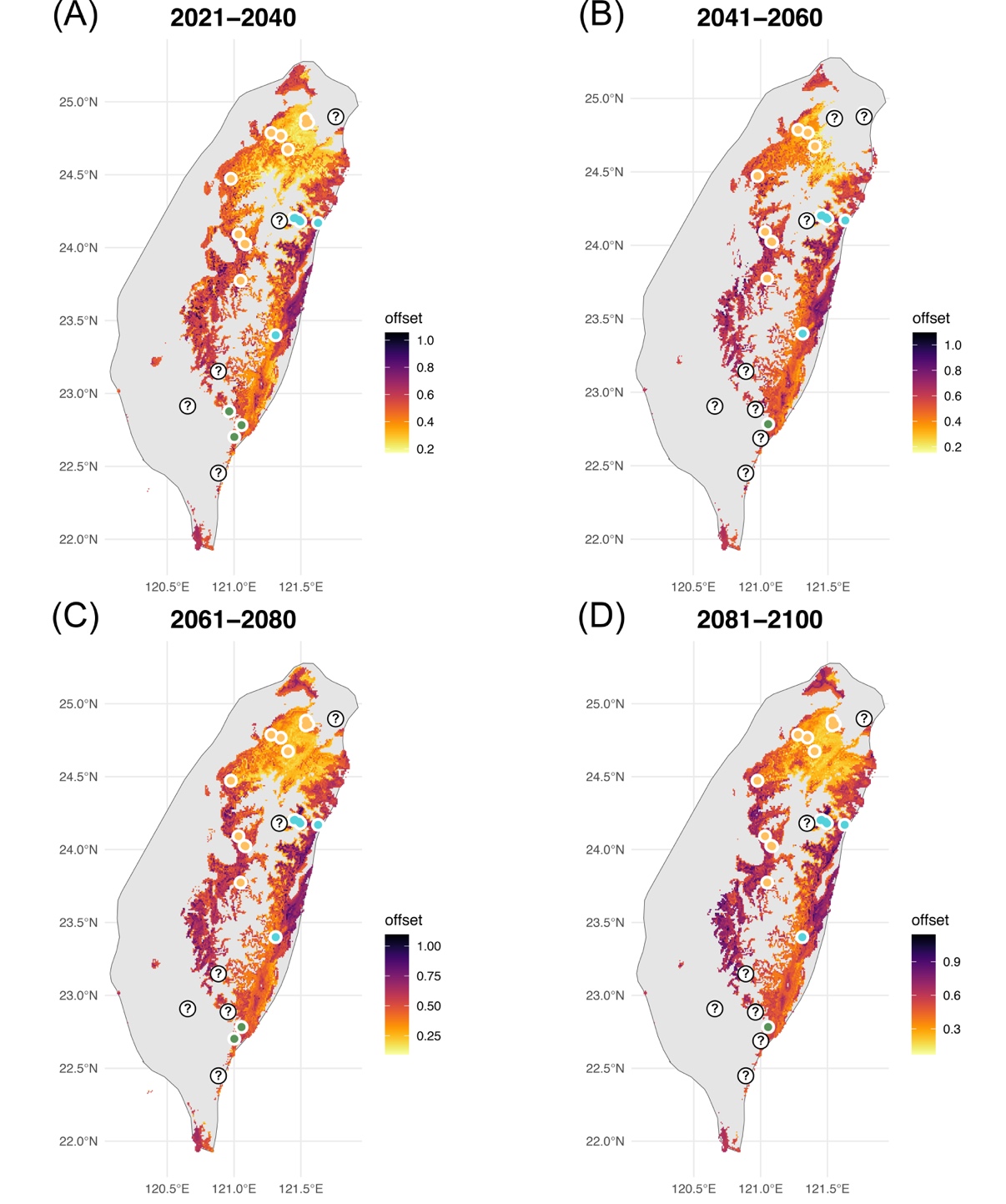


**FIGURE S10 |** Projections of genetic offset under SSP585 for the four lineages of *Euthalia formosana*. Point colors indicate regional lineages: southwest (red), northwest (yellow), southeast (green), and northeast (blue). Genetic offset values represent the magnitude of genomic change required for populations to remain environmentally matched under future conditions, with darker colors indicating greater deviation from current conditions. Populations for which all individuals fall outside the projected future environmental domain used to calibrate the model are marked as non-analog (question mark). Panels show predicted genetic offset for (A) 2021–2040, (B) 2041–2060, (C) 2061–2080, and (D) 2081–2100.

**TABLE S1 |** Number of R80 loci identified under different M/n settings for the full dataset and the *Euthalia formosana*–only dataset.

| **Full dataset** | | | ***Euthalia Formosan*-only dataset** | | |
| --- | --- | --- | --- | --- | --- |
| **Settings** | **R80 loci** | **Difference with previous M/n** | **Settings** | **R80 loci** | **Difference with previous M/n** |
| M1n1 | 15,767 | - | M1n1 | 10,411 | - |
| M2n2 | 15,190 | -577 | M2n2 | 10,630 | 219 |
| M3n3 | 14,520 | -670 | M3n3 | 10,495 | -135 |
| M4n4 | 14,061 | -459 | M4n4 | 10,337 | -158 |
| M5n5 | 13,707 | -354 | M5n5 | 10,176 | -161 |
| M6n6 | 13,476 | -231 | M6n6 | 10,025 | -151 |
| M7n7 | 13,274 | -202 | M7n7 | 9,878 | -147 |
| M8n8 | 13,079 | -195 | M8n8 | 9,745 | -133 |

**Data S1 |** Sampling information and genetic cluster assignments inferred by snapclust. Assignments are shown for K = 3 and K = 4.

| **Individual ID** | **Accession** | **Species name** | **Locality** | **Longititude** | **Latitude** | **Clusters based on K=3** | **Clusters based on K=4** |
| --- | --- | --- | --- | --- | --- | --- | --- |
| N1_0689 | SAMN54621145 | *Euthalia formosana* | TAIWAN: Kaohsiung, Lioguei, Wugongshan | 120.648707 | 22.91355 | Southwest | Southwest |
| N1_0690 | SAMN54621146 | *Euthalia formosana* | TAIWAN: Kaohsiung, Lioguei, Wugongshan | 120.648707 | 22.91355 | Southwest | Southwest |
| N1_0691 | SAMN54621147 | *Euthalia formosana* | TAIWAN: Kaohsiung, Lioguei, Wugongshan | 120.648707 | 22.91355 | Southwest | Southwest |
| N1_0692 | SAMN54621148 | *Euthalia formosana* | TAIWAN: Kaohsiung, Lioguei, Wugongshan | 120.648707 | 22.91355 | Southwest | Southwest |
| N1_0693 | SAMN54621149 | *Euthalia formosana* | TAIWAN: Kaohsiung, Lioguei, Wugongshan | 120.648707 | 22.91355 | Southwest | Southwest |
| N1_0710 | SAMN54621150 | *Euthalia formosana* | TAIWAN: Kaohsiung, Taoyuan, Xiaoguanshan forest road | 120.876123 | 23.15142 | Southwest | Southwest |
| N1_0711 | SAMN54621151 | *Euthalia formosana* | TAIWAN: Kaohsiung, Taoyuan, Xiaoguanshan forest road | 120.876123 | 23.15142 | Southwest | Southwest |
| N1_0712 | SAMN54621152 | *Euthalia formosana* | TAIWAN: Kaohsiung, Taoyuan, Xiaoguanshan forest road | 120.876123 | 23.15142 | Southwest | Southwest |
| N1_0713 | SAMN54621153 | *Euthalia formosana* | TAIWAN: Kaohsiung, Taoyuan, Xiaoguanshan forest road | 120.876123 | 23.15142 | Southwest | Southwest |
| N1_0714 | SAMN54621154 | *Euthalia formosana* | TAIWAN: Kaohsiung, Taoyuan, Xiaoguanshan forest road | 120.876123 | 23.15142 | Southwest | Southwest |
| N1_0715 | SAMN54621155 | *Euthalia formosana* | TAIWAN: Kaohsiung, Taoyuan, Xiaoguanshan forest road | 120.876123 | 23.15142 | Southwest | Southwest |
| N1_2478 | SAMN54621156 | *Euthalia formosana* | TAIWAN: Kaohsiung, Taoyuan, Xiaoguanshan forest road | 120.876123 | 23.15142 | Southwest | Southwest |
| N1_2479 | SAMN54621157 | *Euthalia formosana* | TAIWAN: Kaohsiung, Taoyuan, Xiaoguanshan forest road | 120.876123 | 23.15142 | Southwest | Southwest |
| N1_0733 | SAMN54621158 | *Euthalia formosana* | TAIWAN: Nantou, Renai, Nanshanxi, Menggu waterfall | 121.086015 | 24.022051 | Northwest | Northwest |
| N1_4191 | SAMN54621159 | *Euthalia formosana* | TAIWAN: Hualien, Xiulin, Shangguanyuan | 121.340682 | 24.186516 | Northwest | Northwest |
| N1_0578 | SAMN54621160 | *Euthalia formosana* | TAIWAN: Nantou, Renai, Huisun forest recreation area | 121.033707 | 24.091109 | Northwest | Northwest |
| N1_2484 | SAMN54621161 | *Euthalia formosana* | TAIWAN: Nantou, Xinyi, Danda forest road, Sanfensuo, 950m | 121.049349 | 23.773153 | Northwest | Northwest |
| N1_2687 | SAMN54621162 | *Euthalia formosana* | TAIWAN: Nantou, Xinyi, Danda forest road, Sanfensuo, 950m | 121.049349 | 23.773153 | Northwest | Northwest |
| N1_2824 | SAMN54621163 | *Euthalia formosana* | TAIWAN: Nantou, Renai, Nanshanxi | 121.07962 | 24.02592 | Northwest | Northwest |
| N1_8796 | SAMN54621164 | *Euthalia formosana* | TAIWAN: Miaoli, Taian | 120.976463 | 24.471916 | Northwest | Northwest |
| N1_2485 | SAMN54621165 | *Euthalia formosana* | TAIWAN: Nantou, Xinyi, Danda forest road, Sanfensuo, 950m | 121.049349 | 23.773153 | Northwest | Northwest |
| N1_2844 | SAMN54621166 | *Euthalia formosana* | TAIWAN: Nantou, Renai, Nanshanxi | 121.07962 | 24.02592 | Northwest | Northwest |
| N1_2923 | SAMN54621167 | *Euthalia formosana* | TAIWAN: Nantou, Renai, Nanshanxi | 121.07962 | 24.02592 | Northwest | Northwest |
| N1_8793 | SAMN54621168 | *Euthalia formosana* | TAIWAN: Miaoli, Taian | 120.976463 | 24.471916 | Northwest | Northwest |
| N1_8794 | SAMN54621169 | *Euthalia formosana* | TAIWAN: Miaoli, Taian | 120.976463 | 24.471916 | Northwest | Northwest |
| N1_8795 | SAMN54621170 | *Euthalia formosana* | TAIWAN: Miaoli, Taian | 120.976463 | 24.471916 | Northwest | Northwest |
| N1_4190 | SAMN54621171 | *Euthalia formosana* | TAIWAN: Hualien, Xiulin, Shangguanyuan | 121.340682 | 24.186516 | Northwest | Northwest |
| N1_0728 | SAMN54621172 | *Euthalia formosana* | TAIWAN: Taoyuan, Fuxing, Daman | 121.403827 | 24.673401 | Northwest | Northwest |
| N1_0759 | SAMN54621173 | *Euthalia formosana* | TAIWAN: Taoyuan, Fuxing, Luoma road | 121.278623 | 24.787132 | Northwest | Northwest |
| N1_2896 | SAMN54621174 | *Euthalia formosana* | TAIWAN: New Taipei, Wulai, Honghegu | 121.538739 | 24.884312 | Northwest | Northwest |
| N1_2921 | SAMN54621175 | *Euthalia formosana* | TAIWAN: New Taipei, Wulai | 121.549697 | 24.856876 | Northwest | Northwest |
| N1_2820 | SAMN54621176 | *Euthalia formosana* | TAIWAN: Taoyuan, Fuxing, Northern cross-island highway | 121.350223 | 24.766306 | Northwest | Northwest |
| N1_2951 | SAMN54621177 | *Euthalia formosana* | TAIWAN: New Taipei, Wulai, Baoqing temple | 121.537191 | 24.851207 | Northwest | Northwest |
| N1_2900 | SAMN54621178 | *Euthalia formosana* | TAIWAN: New Taipei, Wulai, Bird watching path | 121.555784 | 24.857261 | Northwest | Northwest |
| N1_2835 | SAMN54621179 | *Euthalia formosana* | TAIWAN: Taoyuan, Fuxing, Daman | 121.403827 | 24.673401 | Northwest | Northwest |
| N1_2486 | SAMN54621180 | *Euthalia formosana* | TAIWAN: New Taipei, Pinglin, Meishan buda temple | 121.763522 | 24.901531 | Northwest | Northwest |
| N1_4379 | SAMN54621181 | *Euthalia formosana* | TAIWAN: New Taipei, Pinglin, Meishan buda temple | 121.763522 | 24.901531 | Northwest | Northwest |
| N1_5521 | SAMN54621182 | *Euthalia formosana* | TAIWAN: Hualien, Xiulin, Luoshao | 121.455523 | 24.203775 | East | Northeast |
| N1_2891 | SAMN54621183 | *Euthalia formosana* | TAIWAN: Hualien, Xiulin, Tarako, Xiangde temple | 121.496133 | 24.179337 | East | Northeast |
| N1_2899 | SAMN54621184 | *Euthalia formosana* | TAIWAN: Hualien, Xiulin, Dali | 121.629201 | 24.169269 | East | Northeast |
| N1_2905 | SAMN54621185 | *Euthalia formosana* | TAIWAN: Hualien, Xiulin, Tarako, Huoranting | 121.485917 | 24.190273 | East | Northeast |
| N1_2916 | SAMN54621186 | *Euthalia formosana* | TAIWAN: Hualien, Xiulin, Tarako, Xiangde temple | 121.496133 | 24.179337 | East | Northeast |
| N1_2930 | SAMN54621187 | *Euthalia formosana* | TAIWAN: Hualien, Xiulin, Tarako, Huoranting | 121.485917 | 24.190273 | East | Northeast |
| N1_2955 | SAMN54621188 | *Euthalia formosana* | TAIWAN: Hualien, Zhuoxi, Zhongping forest road | 121.312168 | 23.399287 | East | Northeast |
| N1_4812 | SAMN54621189 | *Euthalia formosana* | TAIWAN: Hualien, Xiulin, Tarako, Huoranting | 121.485917 | 24.190273 | East | Northeast |
| N1_4974 | SAMN54621190 | *Euthalia formosana* | TAIWAN: Hualien, Xiulin, Tarako, Huoranting | 121.485917 | 24.190273 | East | Northeast |
| N1_2264 | SAMN54621191 | *Euthalia formosana* | TAIWAN: Hualien, Xiulin, Tarako, 153k | 121.447804 | 24.202142 | East | Northeast |
| N1_2956 | SAMN54621192 | *Euthalia formosana* | TAIWAN: Hualien, Zhuoxi, Zhongping forest road | 121.312168 | 23.399287 | East | Northeast |
| N1_2954 | SAMN54621193 | *Euthalia formosana* | TAIWAN: Hualien, Zhuoxi, Zhongping forest road | 121.312168 | 23.399287 | East | Northeast |
| N1_2958 | SAMN54621194 | *Euthalia formosana* | TAIWAN: Hualien, Zhuoxi, Zhongping forest road | 121.312168 | 23.399287 | East | Northeast |
| N1_2957 | SAMN54621195 | *Euthalia formosana* | TAIWAN: Hualien, Zhuoxi, Zhongping forest road | 121.312168 | 23.399287 | East | Northeast |
| N1_2902 | SAMN54621196 | *Euthalia formosana* | TAIWAN: Taitung, Yanping, Hongye | 120.964178 | 22.877164 | East | Southeast |
| N1_5248 | SAMN54621197 | *Euthalia formosana* | TAIWAN: Taitung, Beinan, Lijia forest road | 121.056203 | 22.782737 | East | Southeast |
| N1_2915 | SAMN54621198 | *Euthalia formosana* | TAIWAN: Taitung, Yanping, Hongye | 120.964178 | 22.877164 | East | Southeast |
| N1_0867 | SAMN54621199 | *Euthalia formosana* | TAIWAN: Taitung, Yanping, Hongye | 120.964178 | 22.877164 | East | Southeast |
| N1_2815 | SAMN54621200 | *Euthalia formosana* | TAIWAN: Taitung, Yanping, Hongye | 120.964178 | 22.877164 | East | Southeast |
| N1_2816 | SAMN54621201 | *Euthalia formosana* | TAIWAN: Taitung, Yanping, Hongye | 120.964178 | 22.877164 | East | Southeast |
| N1_2834 | SAMN54621202 | *Euthalia formosana* | TAIWAN: Taitung, Yanping, Hongye | 120.964178 | 22.877164 | East | Southeast |
| N1_2862 | SAMN54621203 | *Euthalia formosana* | TAIWAN: Taitung, Yanping, Hongye | 120.964178 | 22.877164 | East | Southeast |
| N1_2898 | SAMN54621204 | *Euthalia formosana* | TAIWAN: Hualien, Xiulin, Dali | 121.629201 | 24.169269 | East | Southeast |
| N1_2922 | SAMN54621205 | *Euthalia formosana* | TAIWAN: Taitung, Yanping, Hongye | 120.964178 | 22.877164 | East | Southeast |
| N1_2960 | SAMN54621206 | *Euthalia formosana* | TAIWAN: Taitung, Beinan, Jhihben forest road | 121.002348 | 22.701897 | East | Southeast |
| N1_2961 | SAMN54621207 | *Euthalia formosana* | TAIWAN: Taitung, Beinan, Jhihben forest road | 121.002348 | 22.701897 | East | Southeast |
| N1_5251 | SAMN54621208 | *Euthalia formosana* | TAIWAN: Taitung, Beinan, Lijia forest road | 121.056203 | 22.782737 | East | Southeast |
| N1_5203 | SAMN54621209 | *Euthalia formosana* | TAIWAN: Taitung, Beinan, Lijia forest road | 121.056203 | 22.782737 | East | Southeast |
| N1_2833 | SAMN54621210 | *Euthalia formosana* | TAIWAN: Taitung, Daren, Tuban nursing station | 120.886248 | 22.45488 | East | Southeast |
| N1_2829 | SAMN54621211 | *Euthalia formosana* | TAIWAN: Taitung, Daren, Tuban nursing station | 120.886248 | 22.45488 | East | Southeast |
| N1_2832 | SAMN54621212 | *Euthalia formosana* | TAIWAN: Taitung, Daren, Tuban nursing station | 120.886248 | 22.45488 | East | Southeast |
| N1_2830 | SAMN54621213 | *Euthalia formosana* | TAIWAN: Taitung, Daren, Tuban nursing station | 120.886248 | 22.45488 | East | Southeast |
| N1_4351 | SAMN54621214 | *Euthalia thibetana* | China: Yunnan, Weixi, Tacheng | 99.3373 | 27.4675 | Outgroup | Outgroup |
